# Dynamic remodeling of histone modifications in response to osmotic stress in *Saccharomyces cerevisiae*

**DOI:** 10.1186/1471-2164-15-247

**Published:** 2014-03-30

**Authors:** Lorena Magraner-Pardo, Vicent Pelechano, María Dolores Coloma, Vicente Tordera

**Affiliations:** Departament de Bioquímica i Biologia Molecular, Universitat de València, C/Dr. Moliner 50, 46100 Burjassot, València Spain; Genome Biology Unit, European Molecular Biology Laboratory, Heidelberg, Germany

**Keywords:** Histone modification, Chromatin, Epigenetics, Gene regulation, Genome-wide, Transcription, Osmotic stress, ChIP-Chip

## Abstract

**Background:**

Specific histone modifications play important roles in chromatin functions; i.e., activation or repression of gene transcription. This participation must occur as a dynamic process. Nevertheless, most of the histone modification maps reported to date provide only static pictures that link certain modifications with active or silenced states. This study, however, focuses on the global histone modification variation that occurs in response to the transcriptional reprogramming produced by a physiological perturbation in yeast.

**Results:**

We did a genome-wide chromatin immunoprecipitation analysis for eight specific histone modifications before and after saline stress. The most striking change was rapid acetylation loss in lysines 9 and 14 of H3 and in lysine 8 of H4, associated with gene repression. The genes activated by saline stress increased the acetylation levels at these same sites, but this acetylation process was quantitatively minor if compared to that of the deacetylation of repressed genes. The changes in the tri-methylation of lysines 4, 36 and 79 of H3 and the di-methylation of lysine 79 of H3 were slighter than those of acetylation. Furthermore, we produced new genome-wide maps for seven histone modifications, and we analyzed, for the first time in *S. cerevisiae,* the genome-wide profile of acetylation of lysine 8 of H4.

**Conclusions:**

This research reveals that the short-term changes observed in the post-stress methylation of histones are much more moderate than those of acetylation, and that the dynamics of the acetylation state of histones during activation or repression of transcription is a much quicker process than methylation.

**Electronic supplementary material:**

The online version of this article (doi:10.1186/1471-2164-15-247) contains supplementary material, which is available to authorized users.

## Background

Evolution has selected the nucleosome structure as the universal eukaryotic genome organization. From yeast to mammals, the nucleosome structure is practically identical. The protein components of nucleosomes, histones, with a highly conserved amino acid sequence, are subjected to a wide variety of specific and reversible post-translational modifications; i.e., acetylation, methylation, phosphorylation, deimination, ADP-ribosylation, among others (Reviewed in [1]). Besides DNA methylation, the pattern of histone modifications has also been called epigenetic information. It is generally agreed that this information plays important roles in chromatin functions, such as activation or repression of gene transcription or nucleosome assembly during replication. However, the mechanisms by which modifications of histones can participate in these processes, especially in transcription regulation, are not yet known, although two main mechanisms have been proposed. One is that histone modifications can directly affect the nucleosomal structure or the folding properties of chromatin to facilitate DNA accessibility by allowing for *trans*-acting factors to gain access to DNA [2]. In the second one, histone modifications can act as a signaling system by actively participating in the recognition of, and the interaction with, *trans*-acting regulators factors [3–6]. Numerous discoveries made in the past decade support the notion that such modifications regulate transcription through the recruitment of effectors protein complexes (Reviewed in [7]). Obviously, both proposed mechanisms of action are not mutually exclusive.

Chromatin immunoprecipitation (ChIP) assay coupled to microarray analysis of immunoprecipitated DNA (ChIP-Chip) has become an invaluable tool for the *in vivo* mapping of histone modifications genome-wide [8–12]. By way of example, OJ Rando and collaborators [11] analyzed 12 histone modifications at nucleosomal resolution by hybridizing a high-density tiling microarray with mononucleosome fragments obtained after micrococcal nuclease treatment. However, the arrays used in that study covered only a small percentage of the yeast genome. A more complete map of histone modifications, describing five histone H3 methylations; mono-, di- and tri-methylation of lysine 4 (H3K4me1, H3K4me2, H3K4me3), tri-methylation of lysine 36 (H3K36me3) and tri-methylation of lysine 79 (H3K79me3), two histone acetylation; lysine 9 of H3 (H3K9ac) and lysine 14 of H3 (H3K14ac) and hyperacetylated histone H4, has been reported in [12]. The most important results obtained were that H3K9ac, H3K14ac and the hyperacetylation of H4 and also H3K4me3, were found at the promoters and the 5′ end of transcription units, that H3K4me2 and H3K79me3 were more enriched in the middle of genes, and that H3K4me1 and H3K36me3 were found throughout the coding region to peak near the 3′ ends of the transcription units. Moreover, all the histone modifications correlated with transcriptional activity, except for H3K4me1, H3K4me2 and H3K79me3 [12]. However, as previously discussed widely [for example, (see [13, 14]), correlation is not causation, and the mechanism by which these histone modifications become involved in transcription needs to be known. While there are no new genome-wide studies on the acetylation status of histones in yeast, the methylation profiles of histone H3 obtained in [12] have subsequently been corroborated by other authors using higher density chips probes [15–17] or ChIP-seq [18, 19].

Despite the power of ChIP-Chip for mapping global patterns of histone modifications, the above-cited studies generally provide only a static picture of levels of modifications under a given set of conditions. However, in order to understand the role of histone modifications in transcriptional regulation and the causality between the two processes, it is paramount to study both process dynamically. To address this question, we used tiling DNA microarrays to analyze eight genome-wide specific histone modifications; H3K9ac, H3K14ac, acetylation of lysine 8 of H4 (H4K8ac), H3K4me1, H3K4me3, H3K36me3, di-methylation of lysine 79 of H3 (H3K79me2) and H3K79me3; before and after a physiological perturbation (osmotic stress for 10 min) that causes genome-wide transcriptional changes. We selected these acetylation sites because they are representative of the target of the three histone acetyltransferases which have been related to transcription activity; Gcn5p [12, 20, 21], Sas3p [22] and Esa1p [12, 21, 23–25]. Methylation sites were selected because the methylation of the histones in *S. cerevisiae* is carried out by three known histone methyltransferases (Set1p, Set2p and Dot1p) that modify H3K4 [8, 26–31], H3K36 [32] and H3K79 [33–36], respectively. This paper demonstrates that gene repression was accompanied by a dramatic drop in acetylation, mainly at the transcription start site (TSS) and its surroundings, whereas the profiles of methylation hardly changed, and that only slight decreases were observed along the transcribed region, with modifications correlating positively with transcription. Gene activation was accompanied by an increased level of acetylation at the TSS, but it was less intense than that of the deacetylation of repressed genes. Once again, only a moderate increase in the methylations correlating with transcription was noted. We also analyzed, for the first time in *S. cerevisiae,* the genome-wide profile of a histone modification, which has been related to transcription regulation [37]: H4K8ac.

## Results and discussion

### A new genome-wide and comprehensive histone modification map for yeast

**Figure 1:**
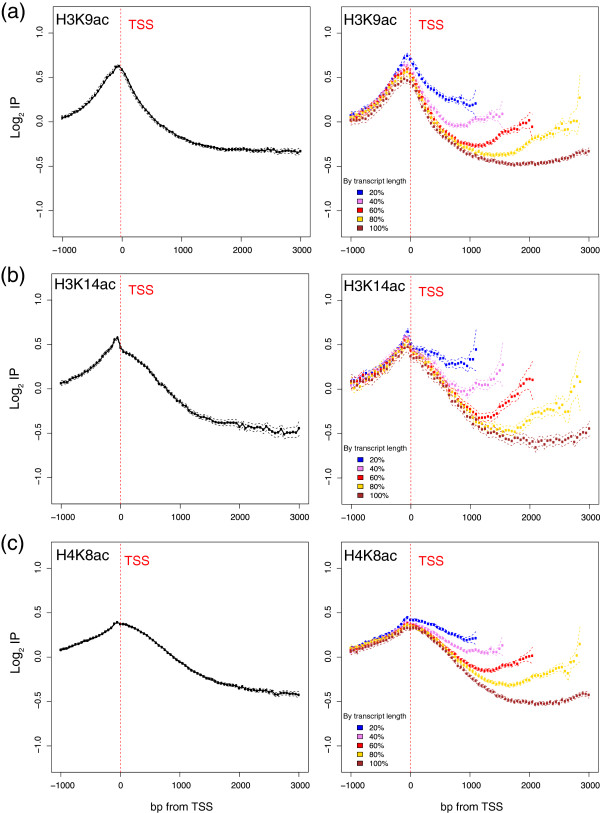
**Genome-wide profiles of histone acetylation.** The levels of H3K9ac **(a)**, H3K14ac **(b)** and H4K8ac **(c)** in relation to the levels of core histone H3 **(a, b)** or H4 **(c)**, genome-wide, are shown. The log2 values of the specific immunoprecipitation of the modified histone were represented in relation to the immunoprecipitation of the core histone (i.e., H3 or H4). For the real-lengths representation (left panels), the profiles were centered on the actual TSS [38] and the data for each probe corresponded to their real distance from the TSS for each gene, from 1000 bp upstream up to the total transcript length, to a maximum of 3000 bp downstream. Probes were binned to 100 bp. The mean and confidence intervals for the means (t-test, 95% confidence) were plotted. Each experiment compared a ChIP with a histone modification antibody to a control ChIP with a core histone antibody. Genes were divided into quintiles according to their transcript length (right panels).

As a first step to study chromatin dynamics, we produced new genome-wide maps for the presence of three histone acetylations under standard growing conditions using high tiling density arrays. The precision of the obtained data allowed us to incorporate important nuances into previous studies. We also analyzed, for the first time in *S. cerevisiae,* the genome-wide profile of H4K8ac. Figure 1 (left panels) shows the profile of the H3K9ac (Figure 1a), H3K14ac (Figure 1b) and H4K8ac (Figure 1c) modifications. We employed a graphical (real-length) representation, where profiles were centered on the actual TSS [38], and the data for each probe corresponded to the distance from the TSS up to the total transcript length, with a maximum of 3000 bp. In this type of representation, the represented number of genes lowered as we moved downstream along the transcribed region. Therefore noise increased, but the structure of the actual profile was maintained. As expected, our general results were consistent with those previously described for H3K9ac and H3K14ac [12]. We also employed a classical representation of genome-wide data using a metagene profile of widespread use [12, 17, 39, 40], where genes are expanded or compressed to fit onto a hypothetical gene of average length (Additional file 1: Figure S1). The comparison made of both types of graphs raised some intriguing questions. For instance, the metagene profile of H3K9ac, H3K14ac and H4K8ac (Additional file 1: Figure S1) shows that these modifications peaked just before the TSS of the genes, and also at the end of the gene. However, when the data were represented in their real length, the increase at the end of the gene disappeared (left panels of Figure 1). To dissect this difference observed between the two representations, we used the real-length representation on sets of genes of different lengths. As the right panels of Figure 1 depict, an actual increase in the signal levels at the 3′ region was observed for all the transcript lengths and acetylation marks, except for the group containing the shortest transcript for the H3K9ac mark and, to a lesser extent, the longer transcript for the three marks. The increase in signal toward the end of the genes was not observed in this representation for long genes as their transcription termination sites are spread from around 2.2 to 15Kb from the TSS and both elongation and terminations marks co-exist at 3Kb. The 3′ accumulation of these marks, typically associated with the start of transcription, suggests that they may also be implicated in the final process stage, or may be caused by neighbor promoters or gene loopings with their own promoter. It is also noticeable that the H3K9ac, H3K14ac and H4K8ac levels were inversely related to their length. The fact that the levels of all these modifications were directly related to transcriptional activity can be explained by the major transcriptional activity of short genes if compared to long genes on average, as previously described [41, 42].

Both H3K9ac and H3K14ac peaked at the probes located between the TSS and −100 bp (Figure 1a,b). This location corresponds precisely to the nucleosome-depleted region (NDR) associated with the TSS [43, 44]. However, as we measured the histone modification levels with the levels of the same unmodified core histone [12], this represented only the relative presence of the modification independently of actual nucleosome abundance. Although the presence of an NDR in the promoters regions is widespread (i.e., affecting mainly TATA-less genes, which represent around 80% of the total), a considerable number of genes exhibited a more variable promoter architecture (Reviewed in [14]). We hypothesized that the poor presence of histone at this position implies that these data are highly influenced by their neighbors; that is, by nucleosomes-1 and +1. In this context, it has been recently proposed that both promoter-bound nucleosomes assume discrete configurations, which not only define the open or closed states of promoters, but distinguish between the “on” and “off” expression states [45]. As the opening and closing of the promoter is a dynamic process, one interesting proposal is that the histone modifications of these nucleosomes are involved in switching between the on and off promoter states.

The H4K8ac profile, which has not been mapped genome-wide in yeast, was similar to that of the above-described H3K9ac and H3K14ac, although the association was not as strong as that observed for H3K9ac and H3K14ac (Figure 1c). Strikingly, the peak of H4K8ac, which we obtained at the TSS, contradicts previously reported results [11], which describe a two-nucleosome hypo-acetylated domain for H4K8, H4K16 and H2BK16 adjacent to the start codons.

We also produced genome-wide maps for H3K4me1, H3K4me3, H3K36me3, H3K79me2 and H3K79me3 (Additional file 2: Figure S2 a-e) under standard growing conditions. As expected, our general results were consistent with those previously described. [12, 15–19].

### Modification of histones in gene clusters

**Figure 2:**
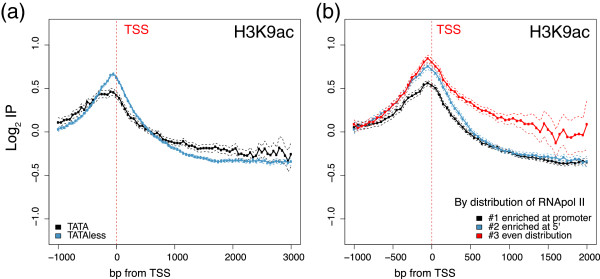
**H3K9ac profiles of a set of genes.** The real-lengths representation of the profile of H3K9ac of the genes in relation to the levels of core histone H3 grouped according to presence or absence of a TATA box **(a)**, or according to the distribution of RNA pol II [46] on the gene **(b)**. The log2 values of the specific immunoprecipitation of the modified histone were represented in relation to the immunoprecipitation of core histone H3.

We also investigated the modifications associated with a particularly interesting set of genes. A well-known example for the groups of genes associated with a specific profile of histone modifications is the presence or absence of the canonical TATA box in their promoter. We represented (Figure 2a and Additional file 3: Figure S3) all the profiles of the modifications of the histones analyzed in this work separately for both the TATA genes and the TATA-less genes. It is remarkable that for all the modifications studied, peaks were higher for the TATA-less genes (as seen in Figure 2a for H3K9ac), with the expected exception of H3K4me1, for which the opposite occurred. The TATA genes are typically stress-responsive and regulated by a variety of chromatin remodeling factors (reviewed in [14]), and this was also the case under our cell growth conditions (exponential growth in rich media) as the median expression of the TATA genes was higher than in the TATA-less ones [41]. Such differences should be due to their different promoter architecture.

In addition to classifying genes according to their promoter structure, BJ Venters and BF Pugh classified them in terms of the differential accumulation of RNA pol II along the promoter and the coding region [46]. Thus, these authors identified three groups of yeast genes in which RNA pol II was enriched at the promoters in relation to the gene body (Group 1), enriched immediately downstream of the promoters (Group 2), or spread throughout the genes (Group 3). These authors suggested that this subgenic location can reflect rate-limiting steps during transcription, including initiation, elongation and termination. We represent the histone modifications profiles for these three groups in Figure 2b and Additional file 4: Figure S4. In order to correctly interpret these graphs, it is important to consider that, on average, the genes of Group 1 were the least active (*i.e.,* smaller total amount of RNA pol II present along the gene), the genes of Group 3 were the most active (*i,e.,* more RNA pol II present along the gene), and that our data were always normalized by core histones. It is not surprising, therefore, that the genes in Group 1 showed a less intense H3K9ac peak at the TSS than the other genes (Figure 2b). Another interesting point is that the Group 3 genes, which displayed a comparatively even distribution of RNA pol II across their genes, or even enrichment toward the 3′ end, showed a clear increase in H3K9ac (Figure 2b), H3K14ac, H4K8ac and H3K79me2 (Additional file 4: Figure S4) in the body of genes if compared to the other genes. This scenario suggests that the presence of RNAPII results in the increased recruitment of HATs and Dot1, which enhance these modifications. It is also noteworthy that the presence of RNA pol II poised at the promoters in the genes of Group 1 was accompanied by an increase in signals H3K4me1 and H3K36me3 at the promoters if compared to the other groups of genes (Additional file 4: Figure S4). It has also been reported that H3K4me1 does not require the ubiquitination of histone H2B, suggesting a Paf1-independent targeting of Set1 to the coding region of active genes [47]. An increased H3K4me1 signal in the promoters with poised RNA pol II could result from this targeting of Set1, while an increase in H3K36me3 suggests that Set2p is possibly present, together with RNA pol II, in this early process step.

### Correlation between histone modifications and the transcription rate, mRNA amount and RNA pol II presence

Transcriptional activity is tightly linked to histone modifications. However, transcriptional activity can be measured by different indicators. The simplest indicator of transcriptional activity is presence of mRNA. Nevertheless, more direct indicators of transcriptional activity, such as presence of RNA pol II in the body of genes [48] or nascent RNA production, are also available [41]. Many researchers have also utilized a data set [49], which represents the indirect calculation of the rate of appearance of mature mRNAs in the cytoplasm. Yet all these measures (i.e., mRNA amount, RNA pol II occupancy, nascent transcription rate and indirect transcription rate) reflect different biological realities. We compared all four data sets with the level of the eight histone modifications studied in both the promoters region and the ORFs in Additional file 5: Table S1. The results indicate that all the analyzed modifications correlated positively with the four data sets employed, except for H3K4me1, which correlated negatively, as expected [12]. Moreover, correlations were always better for RNA pol II occupancy than for the other data sets, and the nascent TR data sets generally correlated better than the indirect data sets, especially with H3K36me3, a chromatin mark that has been directly related with transcription elongation [12]. The worst correlation of all was noted for amounts of mRNA (Additional file 5: Table S1), indicating that post-transcriptional processes, such as mRNA stability, must be taken into account for correct data interpretation [41].

**Figure 3:**
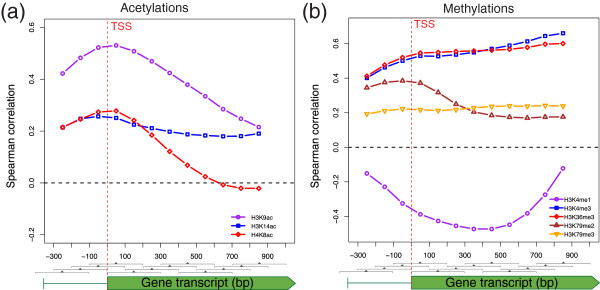
**Correlation between histone modifications and RNA pol II occupancy at different gene locations.** The Spearman rank correlation among the eight histone modifications and RNA pol II occupancy. Gene regions were split using a sliding window of 300 bp along the ORFs (from the promoter regions to the internal regions) Histone acetylations **(a)** and histone methylations **(b)**. The Spearman correlations between whole gene RNApol II occupancy (Rpb3) [48] and local histone modification were computed for each region.

To acquire information about the particular positions at which histone modification can recruit transcription factors or can be modified as a result of transcription, we computed the precise location along the gene where each histone modification better correlated with transcription. Figure 3 shows the Spearman rank correlation between the eight histone modifications and the RNA pol II occupancy obtained at different gene locations. Several important conclusions can be drawn from this figure. First, H3K9ac, H3K14ac and H4K8ac show a profile with a peak centered at the TSS, which decreased downstream. While this figure depicts a less pronounced H3K14ac peak, we performed similar experiments in other yeast strains. The peak obtained for H3K14ac was similar to that of H3K9ac, and was only slightly lower in height (results not shown). Second, H3K4me3 and H3K36me3 exhibited a practically identical profile, and it was not possible to distinguish if one modification preceded the other, or vice versa. H3K79me3 displayed a profile of positive correlations with a similar shape to that of H3K4me3 or H3K36me3, but of less intensity. This result contradicts a previous study which found no correlation between the level of H3K79me3 and transcriptional activity [12]. The discrepancy between these two genome-wide profiling studies in yeast, and also in other eukaryotes, has been attributed to the different chromatin preparation methods used and, more specifically, to the presence or absence of sodium dodecyl sulfate in formaldehyde-cross-linked chromatin [50]. As recently discussed however [49], analyses of mouse, fly, and human genome have revealed that H3K79 methylation is indeed a marker of active transcription, which thus confirms our results in yeast. Finally, as in the gene modifications profiles, H3K79me2 once again showed a very similar profile of positive correlations to those of the histone acetylation peaking at the TSS.

Our results indicate that the histone modifications which better correlate with transcription activity are those of H3K4me3 and H3K36me3 (Spearman correlation of 0.659 and 0.600, respectively), which is in agreement with previous reports [11, 12], and that both modifications can be considered chromatin marks related with transcription elongation (Figure 3). H3K79me3 modifications can also be considered chromatin modifications related with transcription elongation, but of less magnitude. The correlation of H3K4me1 with RNA pol II was negative and gave a high absolute value: −0,473 between +300 and +600 bp in the ORF. According to our results, the most important chromatin modification associated with active promoters was H3K9ac, which reached values of 0.530 between −100 and +200 bp. H3K14ac, H4K8ac and, strikingly, H3K79me2 modifications, can also be considered chromatin modifications in relation to active promoters, but of less magnitude.

### Changes in the modification of histones during the activation or repression of genes

The key question as to the function of histone modifications in transcription regulation is whether or not they are the cause or the consequence of the process itself [1, 13, 14, 51]. In order to gain new insights into this central issue, we studied the histone modifications associated with the drastic change in transcription activity associated with osmotic stress. This is an ideal condition as the molecular mechanism is well-known (reviewed in [52]), and also because independent transcriptomic response data sets are available [53–55]. For our study, we chose the data relating to changes in transcription rate [55] obtained from genomic run-on experiments [56], which offer the chance to study the direct link between transcriptional activity and chromatin (unlike the mRNA abundance data sets which require the accumulation of mRNA molecules). Furthermore, the number of genes that are up- and down-regulated by osmotic stress is large enough to reduce noise and allows the identification of statistically relevant trends. In our case, we used 248 genes defined as being up-regulated and 400 down-regulated genes (Additional file 6: Table S2) [55]. It has also been reported that the activation or repression of osmotic stress genes can be carried out by several mechanisms, which include the recruitment of chromatin-modifying activities (reviewed in [52]). Taken together, these findings suggest that histone modifications can play an important role, at least in the activation of the genes induced by osmotic stress. Finally, osmotic stress is a quick process in which a transcriptional response is obtained in only 10 min [55, 57]. Therefore, we performed a chromatin immunoprecipitation analysis for eight specific histone modifications; H3K9ac, H3K14ac, H4K8ac, H3K4me1, H3K4me3, H3K36me3, H3K79me2 and H3K79me3; before and after osmotic stress (0.4 M NaCl) produced by adding a small volume of a concentrated NaCl solution to the yeast culture 10 min before cross-linking with formaldehyde. Specifically, we measured the histone modification levels in relation to the levels of the same unmodified core histone.

**Figure 4:**
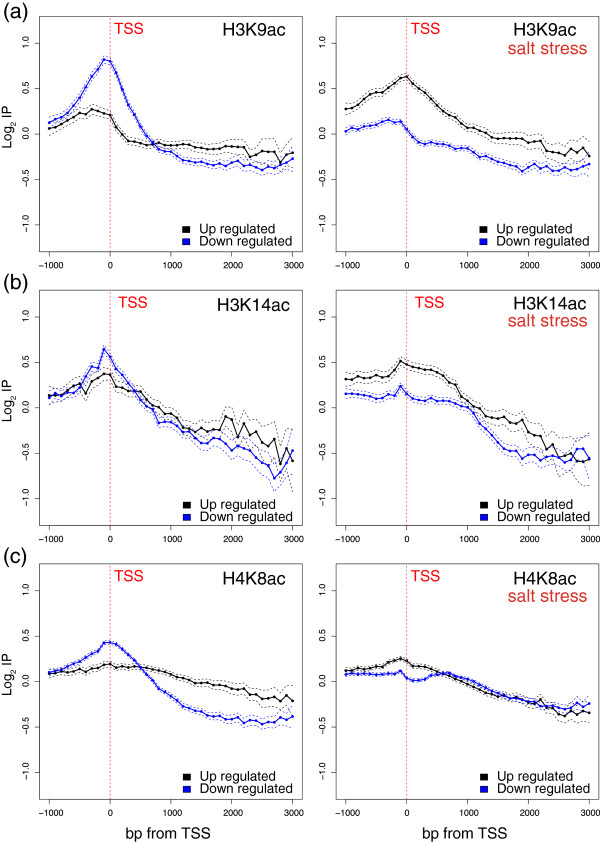
**Genome-wide histone acetylation changes during osmotic stress.** Profiles of H3K9ac **(a)**, H3K14ac **(b)** and H4K8c **(c)** of the genes up- or down-regulated by osmotic stress before (left panels) and after (right panels) stress in relation to the levels of core histones (H3 and H4, respectively). The log2 values of the specific immunoprecipitation of the modified histone were represented in relation to the immunoprecipitation of the core histone (i.e., H3 or H4). The real-lengths representation was presented as in Figure 1.

**Figure 5:**
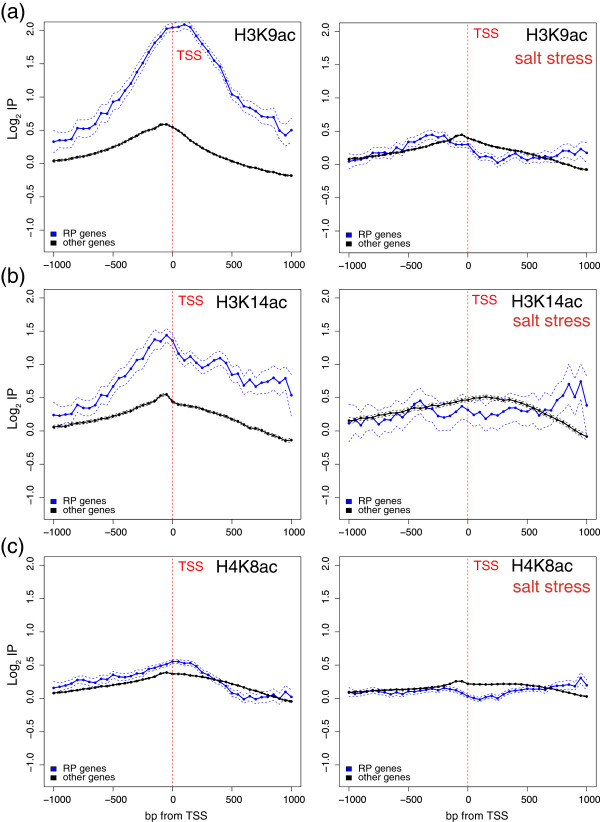
**Histone acetylation changes on RP genes during osmotic stress.** Profiles of H3K9ac **(a)**, H3K14ac **(b)** and H4K8c **(c)** of the RP genes or the remaining genes before (left panels) and after (right panels) osmotic stress in relation to the levels of core histones (H3 and H4, respectively). The log2 values of the specific immunoprecipitation of the modified histone were represented in relation to the immunoprecipitation of the core histone (i.e., H3 or H4). Data were represented as in Figure 4, except that the represented region went from 1000 bp upstream of the TSS to a maximum of 1000 bp downstream. Probes were binned to 50 bp.

The up- and down-regulated genes followed a divergent regulation of the acetylation level upon stress treatment. Figure 4 shows the profiles of H3K9ac, H3K14ac and H4K8c for the genes that were up- or down-regulated in response to osmotic stress before (left panels) and after (right panels) stress. As seen, the up- and down-regulated genes already exhibited an opposite profile before osmotic stress was applied. This is consistent if we consider that the stress-induced genes generally displayed little or no activity before stress and, vice versa, that the genes repressed by stress were more active before stress. The post-stress situation drastically changed. As seen in the right panels, the H3K9, H3K14 and H4K8 acetylations of the down-regulated genes significantly decreased at the TSS and in its surroundings. In fact, the acetylation pattern of the three modifications was similar to that of the stressed up-regulated genes before stress, or was even lower. The profiles of the H3K9, H3K14 and H4K8 acetylations of the genes up-regulated after stress also underwent important changes as their acetylation level at the TSS increased. However, none of the three sites reached the acetylation level of the stressed down-regulated genes before stress. This is perhaps because these genes did not reach a similar transcription rate level to that of the down-regulated genes before stress. These results demonstrate that transcription repression is associated with a quick deacetylation of H3K9, H3K14 and H4K8 at the TSS. To confirm this, we focused on a particular group of genes, ribosomal proteins (RP), which are, with the group of histones, the highest transcribed group of yeast genes under normal growth conditions [41], but are quickly repressed transcriptionally upon osmotic stress [53, 55]. As expected, Figure 5 shows that the H3K14ac and H3K9ac levels of the RP genes were very high before stress (note that the scales in Figures 4 and 5 differ). As seen in the right panels of Figure 5, the acetylation profiles dramatically dropped and reached a similar acetylation level to that of the remaining genes in only 10 min. This result is consistent with the fact that histone acetylation is a rapidly reversible process and that acetyl groups turned over rapidly *in vivo*, with half-lives in the order of minutes [58], thus allowing rapid gene expression changes in response to signals. The H3K9ac profile is especially interesting as it did not follow the same pattern as the remaining genes did because its maximum was presented before stress in the transcribed region in nucleosomes +1 and +2, while other genes displayed their maximum immediately before the TSS. It is precisely in nucleosomes +1 and +2 where the major decrease of H3K9ac took place after stress, suggesting that the H3K9ac of these nucleosomes may play an important role in maintaining the active state of these genes or, alternatively, that its deacetylation is important to switch off their inactivation. Something similar occurred with H4K8ac, but much less intensely. In this context, +1 to +3 nucleosome spacing has been described to be significantly shorter over RP genes than over other gene types, and that polymerase loss results in the relaxation of +1 to +3 spacing in RP genes [59]. It has also been shown that the chromatin structure of RP genes in their 5′ part plays a differential role during transcription elongation [60]. One reasonable hypothesis is that the H3K9ac of these nucleosomes may play a fundamental role in maintaining the special active chromatin architecture of these genes or, alternatively, that its deacetylation is important for them to be switched off, hence their relaxation.

The transcription activation of genes is associated with the acetylation of all these three sites at the TSS. Nevertheless, this process seemed less intense, widespread or slower than that of the deacetylation of the down-regulated genes. Both activities (deacetylation of down-regulated genes and acetylation of up-regulated genes) required the presence of the respective enzymes. Our results cannot explain the reported recruitment of Rpd3 [57], a histone deacetylase involved in deacetylating most core histone sites, except lysine 16 of H4 [61] to osmoresponsive promoters to induce the gene expression of up-regulated genes. In fact, it seems contradictory that the activation of the genes required the recruitment of a histone deacetylase to finally increase its acetylation, unless the sites deacetylated by Rpd3 differed from those analyzed in this study. Conversely, the observation that SAGA is recruited to the osmostres up-regulated genes [62] is in agreement with the increased acetylation level of the genes we found. Gcn5p is a SAGA subunit with histone acetyltransferase activity with specificity for H3K9 and H3K14. Hence, Gcn5p may be responsible for the increase in acetylation at these lysine residues, but is limited to the TSS region. The slight increase in H4K8 acetylation observed may be attributed to another histone acetyltransferase activity specific for this lysine, perhaps Esa1p.

**Figure 6:**
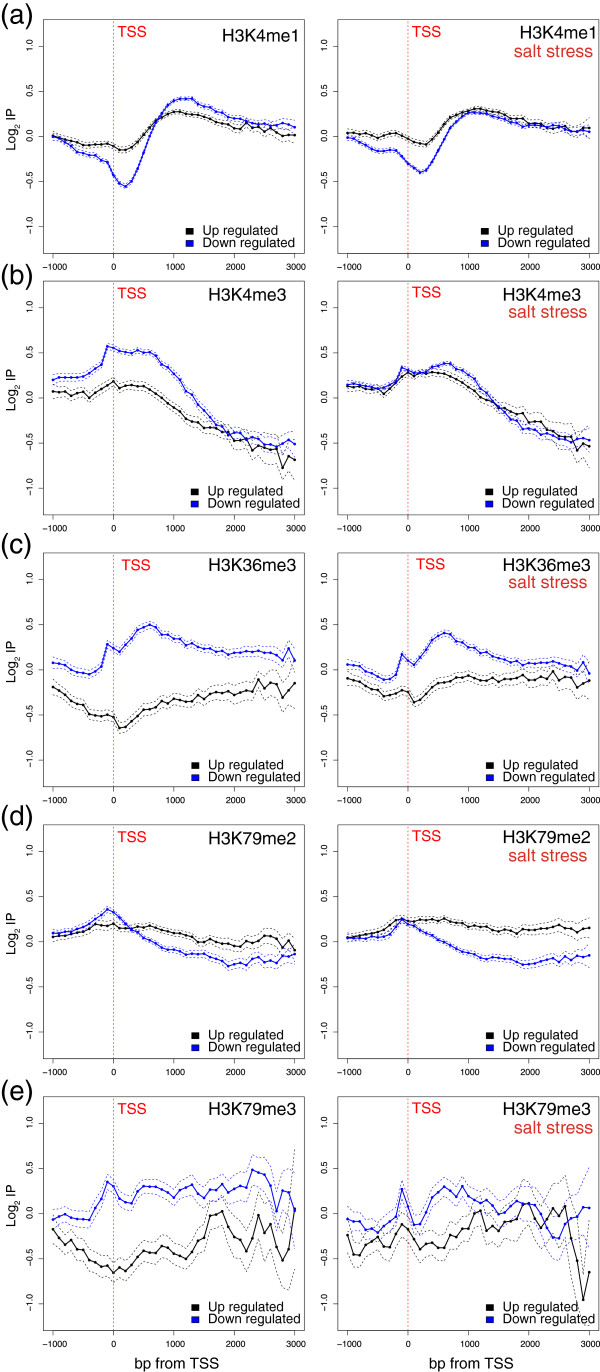
**Genome-wide histone methylation changes during osmotic stress.** Profiles of H3K4me1 **(a)**, H3K4me3 **(b)**, H3K36me3 **(c)**, H3K79me2 **(d)** and H3K79me3 **(e)** of the genes up- or down-regulated by osmotic stress before (left panels) and after (right panels) stress in relation to the levels of core histone H3. The log2 values of the specific immunoprecipitation of the modified histone were represented in relation to the immunoprecipitation of core histone H3. Data were represented as in Figure 4.

**Figure 7:**
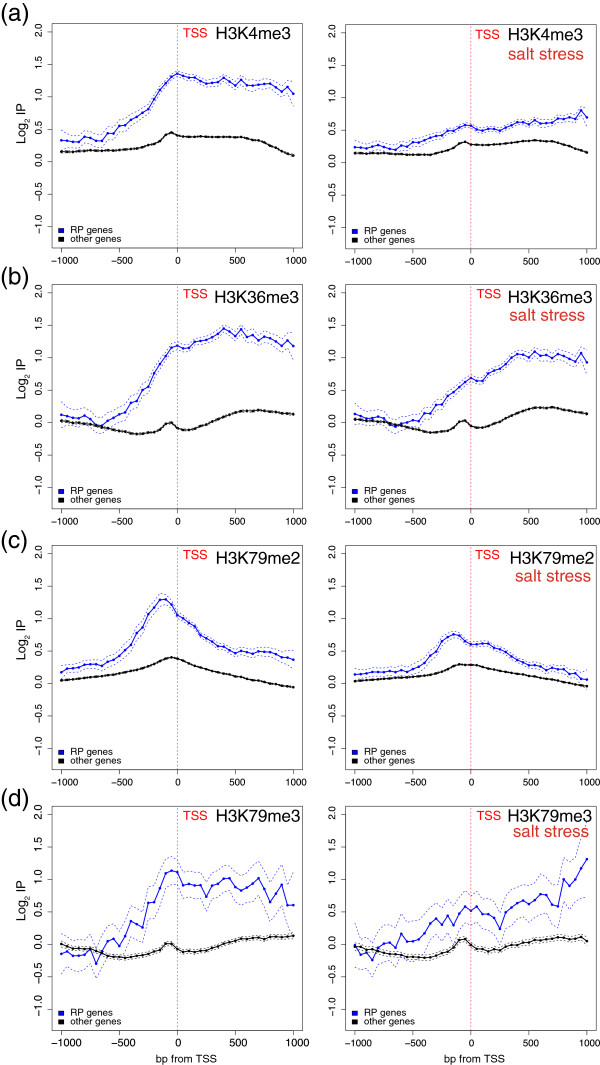
**Histone methylation changes in RP genes during osmotic stress.** Profiles of H3K4me3 **(a)**, H3K36me3 **(b)**, H3K79me2 **(c)** and H3K79me3 **(d)** of the RP genes or the remaining genes before (left panels) and after (right panels) osmotic stress in relation to the levels of core histone H3. The log2 values of the specific immunoprecipitation of the modified histone were represented in relation to the immunoprecipitation of core histone H3. Data were represented as in Figure 5.

Methylation of histone H3 in *S. cerevisiae* has also been related to transcription, mainly H3K4me3 at promoters and H3K36me3 in the coding region [12]. In our study, we measured the changes of the H3K4me1, H3K4me3, H3K36me3, H3K79me2 and H3K79me3 levels brought about by the repression or induction of genes. Figure 6 shows the profile of these histone modifications of genes, which were up- or down-regulated with osmotic stress before (left panels) and after (right panels) stress. Before stress, H3K4me3, H3K36me3, H3k79me2 and H3K79me3 showed a higher modification level in the down-regulated genes than the in the up-regulated ones. With H3K4me1, in which a negative correlation has been reported [12], the reverse effect occurred. Yet the most remarkable point of this experiment was the comparison made of the profiles before and after stress. The methylation levels of the analyzed lysine residues did not change drastically, quite unlike what happened in acetylation. Thus, the methylation profiles of the up- or down-regulated genes barely changed after 10 min of stress, with only slight increases found in the up-regulated genes and slight decreases in the down-regulated genes noted along the transcribed region in H3K4me3, H3K36me3, H3k79me2 and H3K79me3. It is also worth noting that these changes were not limited to either the TSS in H3K4me3 or the transcribed region in H3K36me3, but that both changes occurred in the promoter and the wide region of the 5′ transcribed region. H3K79me2 displayed a particular behavior because the changes caused by stress, mainly in the repression of the down-regulated genes, were located principally near the TSS. It seems that the profiles of both the up- and down-regulated genes tended to generally approach each other after stress. Taken together, these results strongly suggest that the tri-methylation of H3K4, H3K36 and H3K79 across the transcribed region is a process that most likely stems from the presence or passage of RNA pol II, as described by other authors. To determine whether the reverse process, that is, the de-methylation of these lysine residues during the repression of the down-regulated genes, was slow or fast, we re-focused on RP genes. Figure 7 shows the profiles of H3K4me3, H3K36me3, H3K79me2 and H3K79me3 of the RP genes as compared with the remaining genes both before (left panels) and after (right panels) stress. Once again, we observe that all four modifications were very intense before stress (left panels). Repression of these genes by stress lowered all the modifications but, in this case, not at the level of the remaining genes. Furthermore, H3K4 and H3K79 seemed to be de-methylated more intensely or, perhaps, more rapidly than H3K36. Alternatively, it was possible to replace the methylated histones with other non methylated ones in a process mediated by chromatin remodeling complexes.

While this manuscript was being prepared, a genome-wide mapping of five histone modifications, including H3K4me3, H3K36me3 and H3K14ac, during diamide stress was published [63]. Our results agree with the genome-wide changes described in this recently published study. However, these authors also found that the H3K4me3 levels increased at the 5′ ends of a substantial number of diamide-repressed genes during their repression, including RP genes [63], which clearly contradicts our results. The distinct chromatin changes observed in the RP genes during repression can be explained only if both osmotic and diamide stresses operate via distinct pathways.

The timing of the acetylation and methylation processes, shown herein, suggests some major consequence. Thus, several complexes with histone acetyltransferase activity, such as NuA3 [64] or SAGA [65], have been described to bind during gene activation to H3K4me3 to promote their histone acetyltransferase activity toward H3K14. Given our results, it is difficult to accept that the (barely perceptible) changes in the levels of H3K4me3 produced during the activation of transcription in the up-regulated genes can mediate the downstream effects affecting the acetylation state of H3 in a short time when the opposite is much more likely. Consistently with our results, the mutation of histone H3K14 has been reported to result in a loss of H3K4me3 in bulk histones [66], or more recently, that histone H3K4 demethylation is negatively regulated by H3K14 acetylation in *Saccharomyces cerevisiae*[67].

## Conclusions

Our mapping data of the modifications occurring under standard growing conditions generally agree with previous studies [12], although we also observed several unexpected behaviors (i.e., an increase in the H3K9ac, H3K14ac, H4K8ac and H3K79me2 signal levels at the 3′ region with longer genes). In fact, it is strikingly that all the genome-wide features of H3K79me2 described herein are similar to those of acetylation.

To date, most chromatin state maps have provided static pictures of histone modifications. However, the participation of modifications of histones in regulating gene expression must occur as a dynamic process. In this research, we have conducted a study that has focused on the genome-wide changes of several relevant histone modifications associated with the major transcriptional reprogramming caused by osmotic stress. We report herein that the most striking change is the quick deacetylation of H3K9, H3K14 and H4K8, associated with the repression of genes. Activated genes increase the acetylation levels at these same sites, but the acetylation process of activated genes seems smaller in quantitative terms to that of the deacetylation of repressed genes. The changes noted in the acetylation state of osmoregulated genes are caused mainly in the transcription start site (TSS) region. As expected, the tri-methylation of H3K4, H3K36 and H3K79, and also the di-methylation of H3K79, of the activated or repressed genes also changed by increasing and decreasing, respectively. However, the short-term changes observed in the post-stress methylation of histones are much more moderate than those of acetylation. This research work, besides that recently reported while preparing this manuscript [63], is the first genome-wide study of dynamic changes in histone modifications in response to global transcriptional reprogramming in yeast. The observed changes support the acetylation hypothesis given the possibility of them acting as signals involved in triggering the process of activating or repressing transcription. The moderate changes noted in the methylation of histones seem to be a process that occurs as a result of presence of RNA pol II. Our results also indicate that the acetylation state of histones during transcription activation or repression is a much quicker process than methylation.

Here we show that dynamic studies need to be done to gain a better understanding of the relationship between chromatin and transcription. We foresee that an understanding of chromatin will strongly benefit from an even more detailed study into the dynamics of chromatin modifications.

## Methods

### Yeast strain and antibodies

The *S. cerevisiae* strain used in this study for the genome-wide location analysis was BY4742, derived from S288C.

The experiments described in this study compared ChIP with a histone modification antibody to a control ChIP with a core histone antibody. The antibodies (obtained from rabbit, ChIP Grade) recognizing the histone modifications were: α-H3K9ac (Upstate, 06–942), α-H3K14ac (Upstate, 07–353), α-H3K4me1 (Abcam, 8895), α-H3K4me3 (Abcam, 8580), α-H3K36me3 (Abcam, 9050), α-H3K79me2 (Abcam, 3594), α-H3K79me3 (Abcam, 2621), and α-H4K8ac (Abcam, 15823). The antibodies used in the control channel in the ChIP-Chip procedure were α-H3 (Abcam, 1791) for the modifications of the histone H3 experiments and α-H4 (Abcam, 10158) for the modifications of the histone H4 experiments.

### Chromatin immunoprecipitation and osmotic stress

We followed a protocol based on a previously described method [68]. A volume of 40 mL YPD culture from each one (O.D.600 ≈ 0.5-0.8) was set aside, and proteins were cross-linked to their target sites *in vivo* by adding formaldehyde at a final concentration of 1%. Cells were incubated for 30 min at room temperature with occasional mixing, and cross-linking was quenched by adding glycine at a final concentration of 125 mM. Cells were washed 3 times with 30 mL of ice-cold PBS buffer (140 mM NaCl, 2.7 mM KCl, 10 mM Na_2_HPO_4_, 1.8 mM KH_2_PO_4_, pH 7.4). Pelleted cells were processed immediately or were frozen and stored at −80°C for no longer than 1 week. Cells were thawed on ice and resuspended in 300 μL of lysis buffer (50 mM HEPES-KOH pH 7.5, 140 mM NaCl, 1 mM EDTA, 1% Triton X-100, 0.1% sodium deoxycholate, 1 mM Phenylmethylsulfonyl fluoride (PMSF), 1 mM benzamidine and 1 pill of protease inhibitor cocktail (Roche) was dissolved in every 25 mL of buffer). The equivalent of 0.2 mL of frozen glass beads (425–600 mm, Sigma) was added to the cellular suspension and cells were lysed at 4°C for 45 min of vortexing in a Genie 2 vortex with Turbo mix at maximum power. The tube was bored with an incandescent needle at the bottom and placed over another eppendorf tube, and was centrifuged for a few seconds. Beads were washed with a further 400 μL amount of lysis buffer. A final volume of approximately 700 μL of extract was obtained. Sonication of DNA was performed to obtain DNA fragments of between 200–1000 bp, with an average size of 400 bp. Chromatin was sonicated on ice, 8 pulses of 30 s at an amplitude of 38% in a Vibracell VCX500 (Sonics&Materials). Cell debris was removed by centrifugation at 13000 rpm at 4°C for 10 min. An 50-μL aliquot f this whole cell extract was kept to check the quality and size of the isolated chromatin. The antibodies used were 15 μg of rabbit α-histone modified or 20 μg of rabbit α-histone unmodified. Rabbit antibody complexes were collected using 50 μL of a suspension of sheep α-rabbit IgG M-280 dynabeads (Dynal, Invitrogen Corp., Carlsbad, CA, USA) in a final volume of 120 μL. Suspensions were incubated overnight at 4°C. The DNA fragments specifically cross-linked to the antibody were purified by immunoprecipitation with the indicated monoclonal antibodies coupled to dynabeads for 4 h at 4°C. Beads were washed twice with lysis buffer, twice with 500 mM of NaCl lysis buffer, twice with wash buffer (10 mM Tris–HCl pH 8.0, 250 mM LiCl, 0.5% Nonidet P-40, 0.5% sodium deoxycholate, 1 mM EDTA pH 8.0, 1 mM PMSF, 1 mM benzamidine and 1 pill of protease inhibitor cocktail/25 mL from Roche), once with TE containing 1 mM PMSF, and were finally collected. Two successive elutions were performed with 100 and 150 μL of elution buffer (50 mM Tris–HCl pH 8.0, 10 mM EDTA, 1% SDS) by incubating 10 min at 65°C each time. The eluted fraction of the protein cross-linked to DNA was treated overnight at 65°C to reverse the cross-linking.

Osmotic stress (0.4 M NaCl) was produced by adding a small volume of concentrated NaCl solution to the yeast culture 10 min before cross-linking with formaldehyde.

### DNA purification and annealed linkers ligation

Proteins were degraded by adding 50 μg of proteinase K and SDS at a final concentration of 2.5% with incubation at 37°C for 1 h. DNA was purified by phenol/chloroform/isoamylic alcohol extraction. The aqueous phase was subsequently purified with Montage PCR columns (Millipore). The total eluted volume was treated with 10 μg of RNAse A for 30 min at 37°C. Ethanol precipitation was performed in the presence of 20 μg of glycogen as a carrier. Immunoprecipitated DNA was blunted by T4 phage DNA polymerase in a reaction volume of 124 μL (T4 DNA Pol buffer, 40 μg/μL BSA, 80 μM dNTPs, 0.6 U T4 DNA Polymerase from Roche). The reaction was allowed to proceed for 20 min at 12°C. After phenol/chloroform/isoamylic alcohol extraction, DNA was ethanol-precipitated in the presence of 11 μg of glycogen and was ligated in a final volume of 50 μL with annealed linkers oJW102 and oJW103 (1.5 μM of each primer). The reaction was carried out overnight at 16°C and ligated DNA was precipitated.

### DNA amplification and microarray hybridization

Ligation-Mediated PCR was used for DNA amplification [68]. All the immunoprecipitated DNA was used for the LM-PCR reaction. Briefly, ligated DNA was dissolved in final volume of 40 μL (1x Biotaq buffer from Bioline, 2 mM MgCl2, 0.25 mM dNTPs, 1.25 μM oligonucleotide oJW102). The reaction was started by incubating for 2 min at 55°C and was paused to add 10 μL of the reaction mix (1X Biotaq buffer, 2 mM MgCl2 and 5 U BioTaq from Bioline). The program was resumed as so: 5 min at 72°C, 2 min at 95°C and 33 cycles of 30 s at 95°C, 30 s at 55°C and 2 min at 72°C. A 5-μL DNA aliquot of the LM-PCR was analyzed on a 1.2% agarose gel to check for a smear and an average size of 150 bp. The rest was purified with Montage PCR columns. DNA was precipitated overnight and resuspended in 25 μL of milliQ water. Amplified samples were quantified and purity was confirmed by spectrometry (NanoDrop ND1000, NanoDrop Technologies, Wilmington, Delaware, USA). The expected fragment size was confirmed in a DNA 7500 2100 Bioanalyzer (Agilent Technologies, Palo Alto, California, USA) assay. DNA labeling was obtained following the ‘Agilent Yeast ChIP-on-chip Analysis’ protocol, version 9.2 (Agilent Technologies, Palo Alto, California, USA, p/n G4493-90010). Then 2000 ng of ChIP and reference IP (core histone H3 or H4) samples were respectively labeled with Cyanine 5-dUTP and cyanine 3-dUTP using the ‘CGH Labeling Kit’ (Invitrogen, p/n 18095–011) following the manufacturer’s instructions. Labeled DNA was hybridized to the Yeast Whole Genome ChIP-on-chip Microarray (Agilent p/n G4491A, AMADID: 014741), specifically designed for location analyses. The average probe spatial resolution was ~50 nt and it contained ~85% of the non repetitive portion of the yeast genome sourced from yeast sacCer1 (~12 MB). Arrays were scanned in an Agilent Microarray Scanner (Agilent G2565BA) in accordance with the manufacturer’s protocol. Data were extracted using the Agilent Feature Extraction Software, v10.10.1.1, following the Agilent protocol ChIP_1010_Sep10, grid template 014741_D_F_20091202 and metric set ChIP_QCMT_Sep10.

### Data analysis

Data were processed by ChIP Analytics 1.3 (Agilent). Blank subtraction normalization, inter-array median normalization and intra-array median normalization were performed. Probes were lifted over to the sacCer3 genome version (April2011). Transcript boundaries [38] were downloaded from SGD (R64-1-1, http://www.yeastgenome.org). Raw and processed data were deposited at GEO (reference GSE41587).

Plots were performed using R and bioconductor [69]. Probes were binned to 50 bp or 100 bp, and the mean and confidence intervals for the means (t-test 95% confidence) were plotted. For the metagene representation, each transcript was forced to a virtual length of 1000 bp, and a 100 bp bin was applied.

## Electronic supplementary material

Additional file 1: Figure S1: The profiles of H3K9ac **(a)**, H3K14ac **(b)** and H4K8ac **(c)** in relation to the levels of core histone H3 (a, b) or H4 **(c)** genome-wide using a metagene representation. The log2 values of the specific immunoprecipitation of the modified histone were represented in relation to the immunoprecipitation of core histone H3. For the metagene representation, each transcript was forced to a virtual length of 1000 bp, and a 100 bp bin was applied, while the data relating to the 5′UTR and 3′UTR regions corresponded to actual distances, each with a 100-bp unit. The mean and confidence intervals for the means (t-test, 95% confidence) were plotted. Each experiment compared a ChIP with a histone modification antibody to a control ChIP with a core histone antibody. (PDF 210 KB)

Additional file 2: Figure S2: The profiles H3K4me1 **(a)**, H3K4me3 **(b)**, H3K36me3 **(c)**, H3K79me2 **(d)** and H3K79me3 **(e)** in relation to the levels of core histone H3, genome-wide. The log2 values of the specific immunoprecipitation of the modified histone were represented in relation to the immunoprecipitation of core histone H3. Data were represented as in Figure 1. (PDF 2 MB)

Additional file 3: Figure S3: The profiles of H3K14ac **(a)**, H4K8ac **(b)**, H3K4me1 **(c)**, H3K4me3 **(d)**, H3K36me3 **(e)**, H3K79me2 **(f)** and H3K79me3 **(g)** in relation to the levels of core histone H3 **(a, c, d, e, f, g)** or H4 **(b)** of the genes grouped according to presence or absence of a TATA box on the gene. (PDF 1 MB)

Additional file 4: Figure S4: The profiles of H3K14ac **(a)**, H4K8ac **(b)**, H3K4me1 **(c)**, H3K4me3 **(d)**, H3K36me3 **(e)**, H3K79me2 **(f)** and H3K79me3 **(g)** in relation to the levels of core histone H3 **(a, c, d, e, f, g)** or H4 **(b)** of the genes grouped according to the distribution of RNA pol II [46] on the gene. (PDF 1 MB)

Additional file 5: Table S1: Compares the mRNA amount, indirect transcription rate, nascent transcription rate and RNA pol II occupancy data with the level of the eight histone modifications studied in both the promoters region and ORFs. (XLSX 83 KB)

Additional file 6: Table S2: The 248 up-regulated and the 400 down-regulated osmo-responsive genes. (XLSX 39 KB)

## Abbreviations

ChIP: Chromatin immunoprecipitation
ChIP-Chip: Chromatin immunoprecipitation coupled to microarray detection of DNA immunoprecipitated
H3K4me1: Lysine 4 of histone H3 mono-methylated
H3K4me2: di-methylated
H3K4me3: tri-methylated
H3K36me3: Lysine 36 of histone H3 tri-methylated
H3K79me2: Lysine 79 of histone H3 di-methylated
H3K79me3: tri-methylated
H3K9ac: Lysine 9 of histone H3 acetylated
H3K14ac: Lysine 14 of histone H3 acetylated
H4K8ac: Lysine 8 of histone H4 acetylated
TSS: Transcription start site
NDR: Nucleosome depleted region
ORF: Open reading frame
SAGA: Spt–Ada–Gcn5 acetyltransferase
RNA pol II: RNA polymerase II
RP: Ribosomal proteins
NDR: Nucleosome depleted region
CTD: Carboxy terminal domain
PMSF: Phenylmethylsulfonyl fluoride.
