# Supplementary figures and images for: Dynamic remodeling of histone modifications in response to osmotic stress in Saccharomyces cerevisiae
Source: BMC Genomics. 2014 Mar 30;15(1):247. doi: 10.1186/1471-2164-15-247 (PMC3986647; doi:10.1186/1471-2164-15-247)

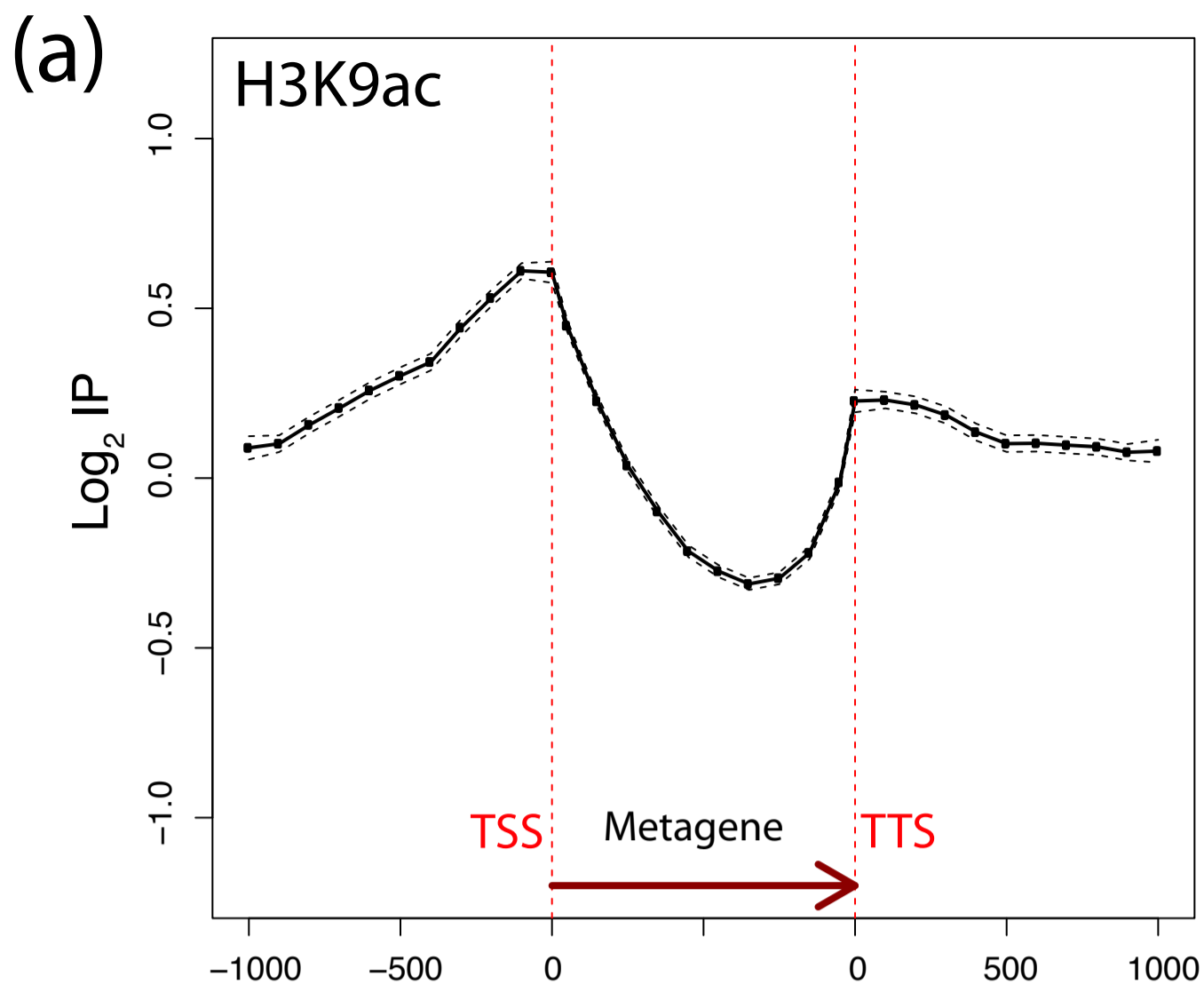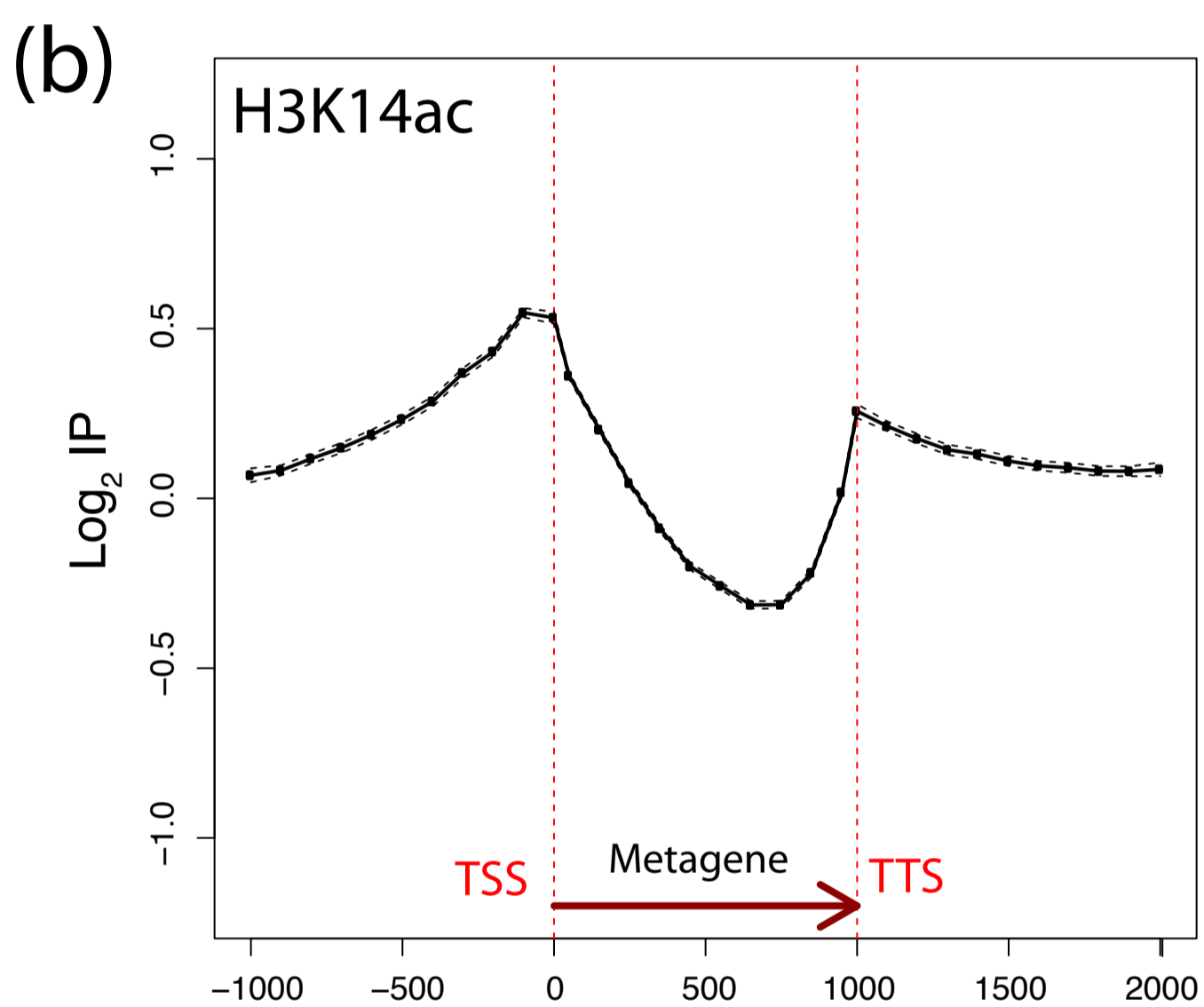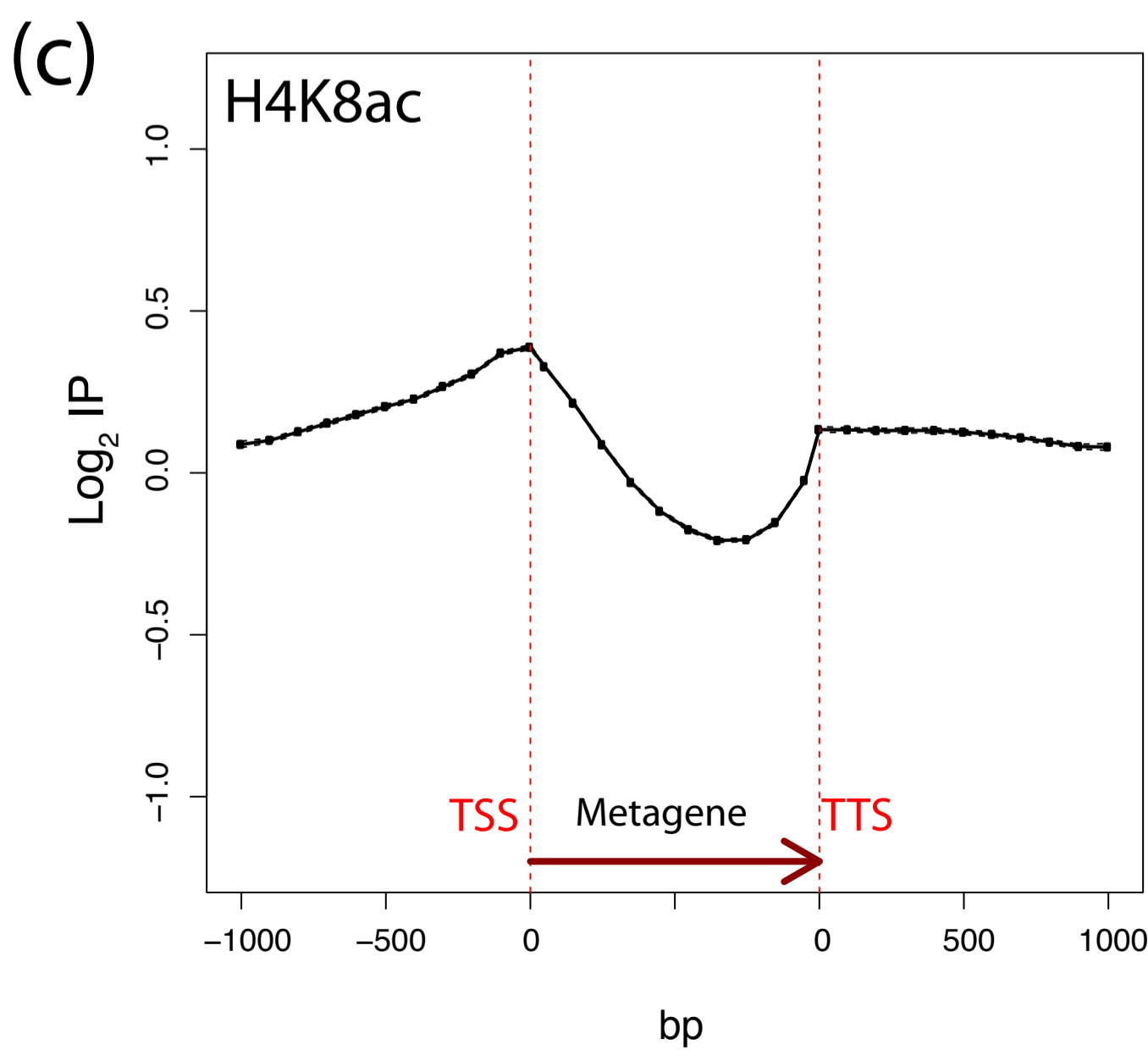

Supplement: Supplementary file 1 — Additional file 1: Figure S1: The profiles of H3K9ac (a), H3K14ac (b) and H4K8ac (c) in relation to the levels of core histone H3 (a, b) or H4 (c) genome-wide using a metagene representation. The log2 values of the specific immunoprecipitation of the modified histone were represented in relation to the immunoprecipitation of core histone H3. For the metagene representation, each transcript was forced to a virtual length of 1000 bp, and a 100 bp bin was applied, while the data relating to the 5′UTR and 3′UTR regions corresponded to actual distances, each with a 100-bp unit. The mean and confidence intervals for the means (t-test, 95% confidence) were plotted. Each experiment compared a ChIP with a histone modification antibody to a control ChIP with a core histone antibody. (PDF 210 KB) [file 12864_2013_5905_MOESM1_ESM.pdf]

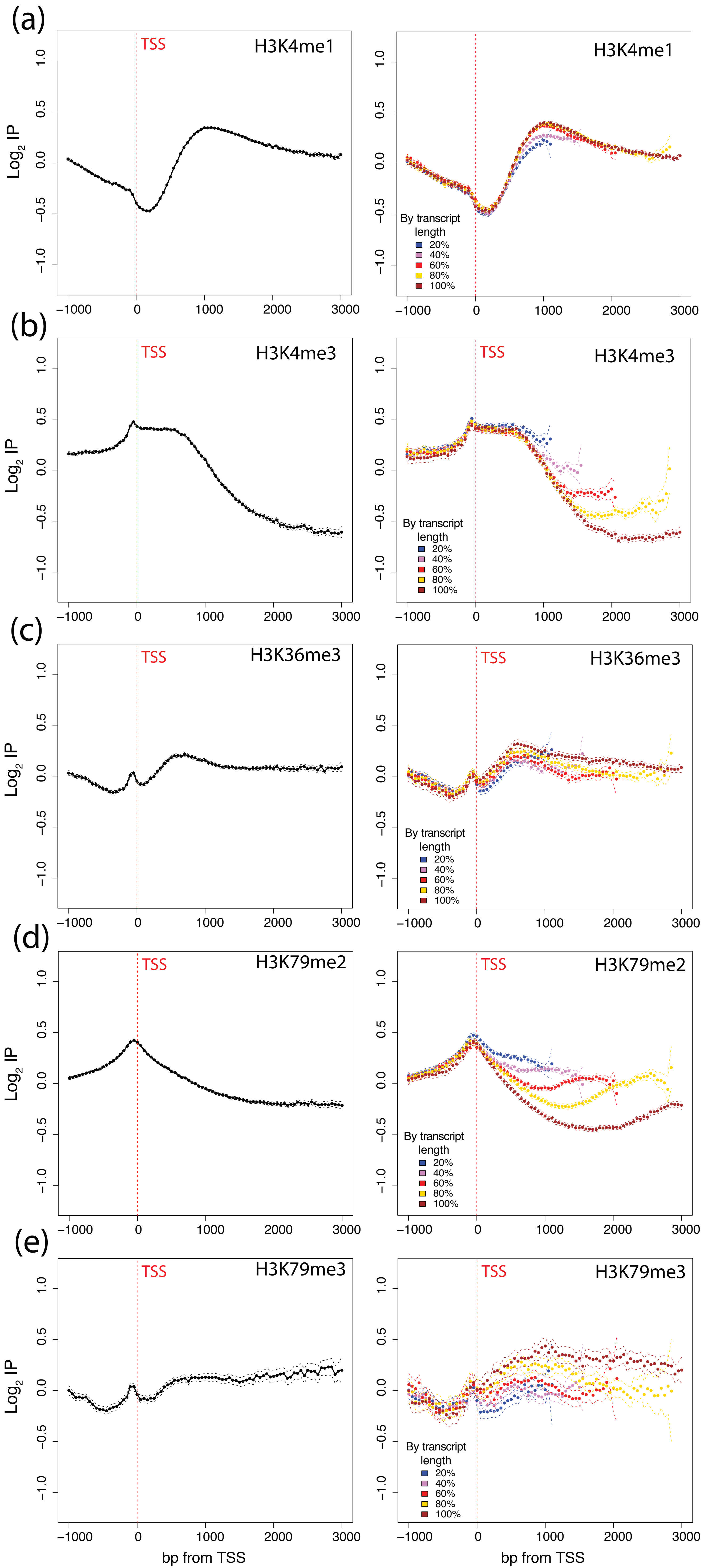

Supplement: Supplementary file 2 — Additional file 2: Figure S2: The profiles H3K4me1 (a), H3K4me3 (b), H3K36me3 (c), H3K79me2 (d) and H3K79me3 (e) in relation to the levels of core histone H3, genome-wide. The log2 values of the specific immunoprecipitation of the modified histone were represented in relation to the immunoprecipitation of core histone H3. Data were represented as in Figure 1. (PDF 2 MB) [file 12864_2013_5905_MOESM2_ESM.pdf]

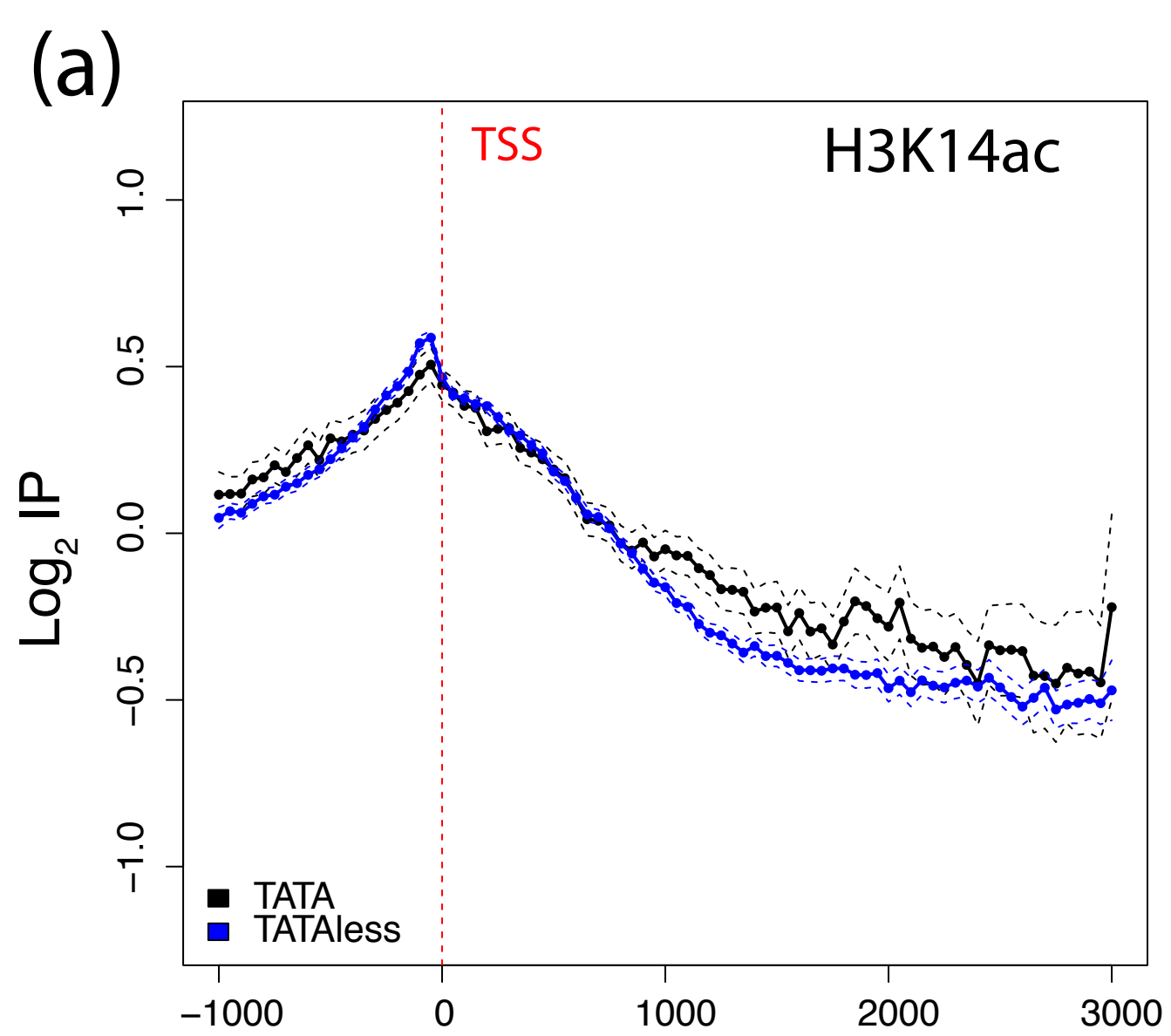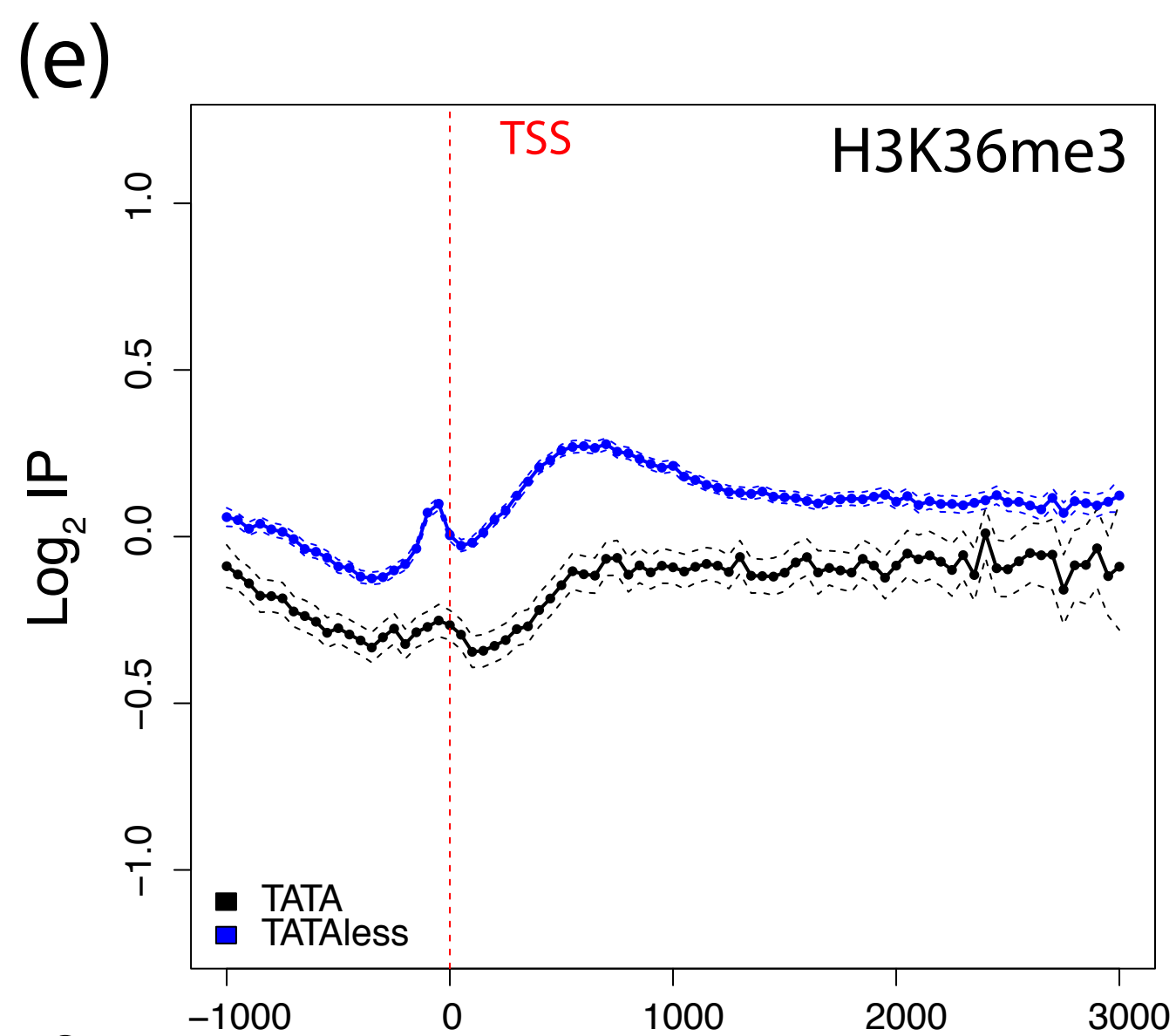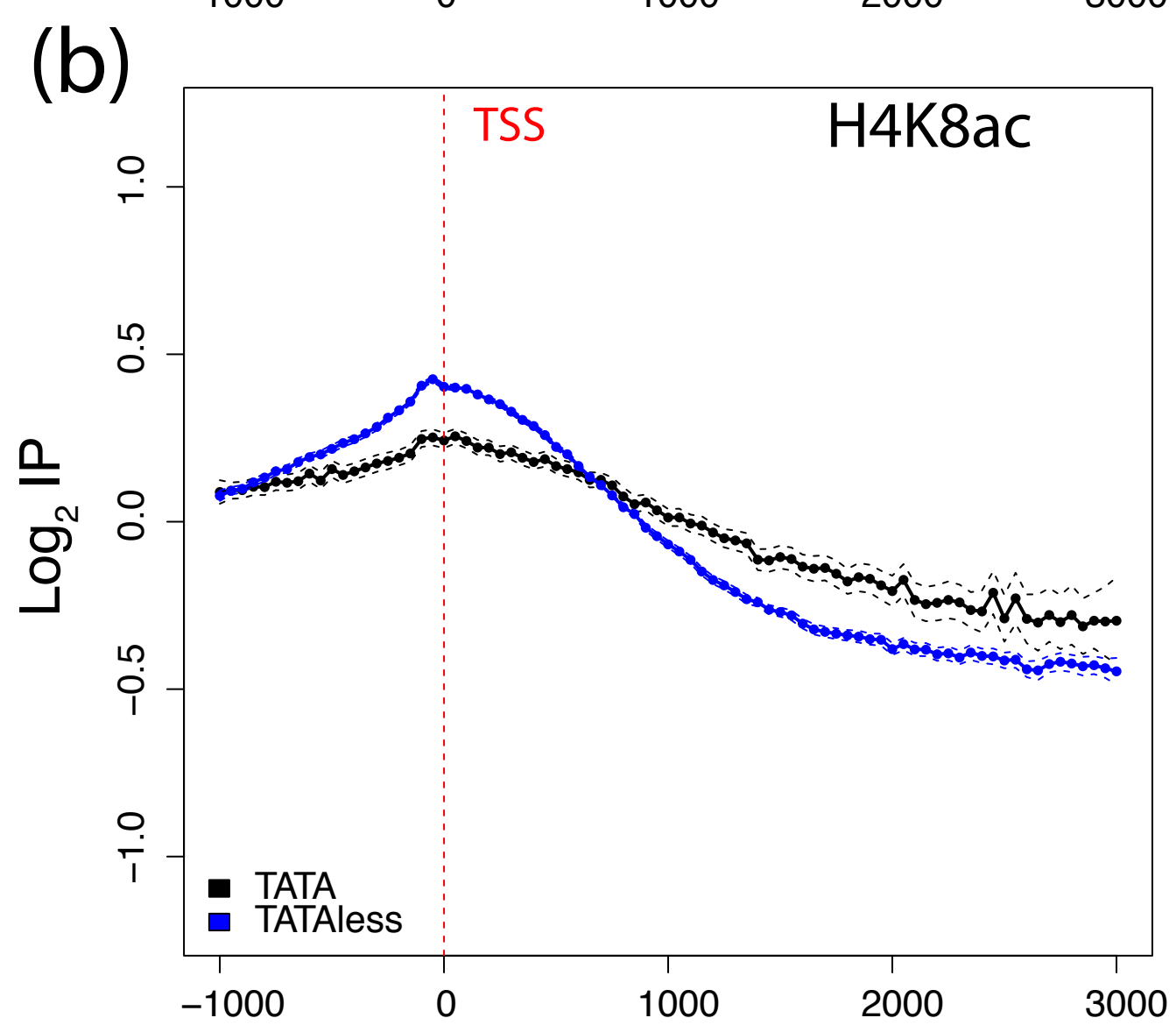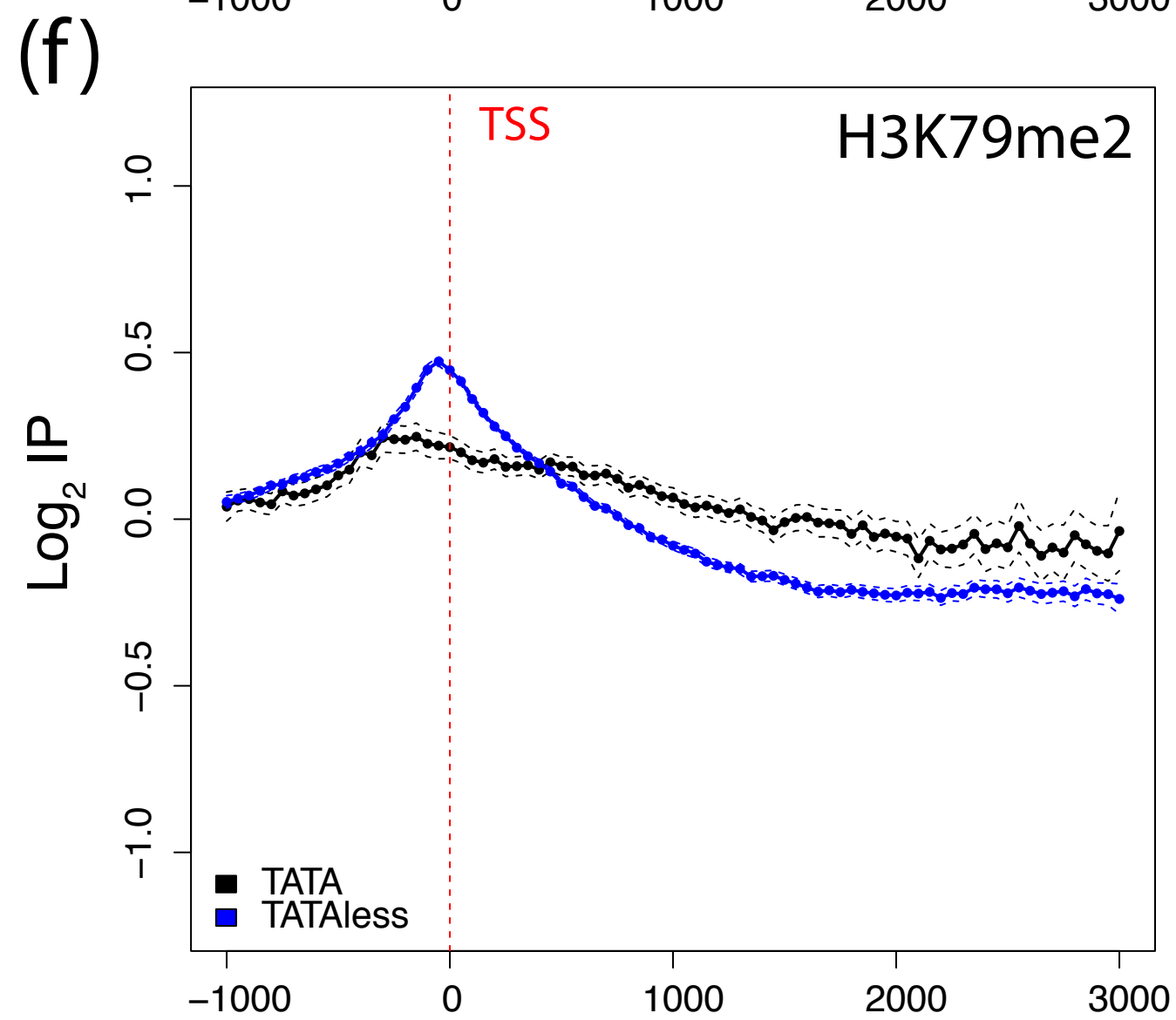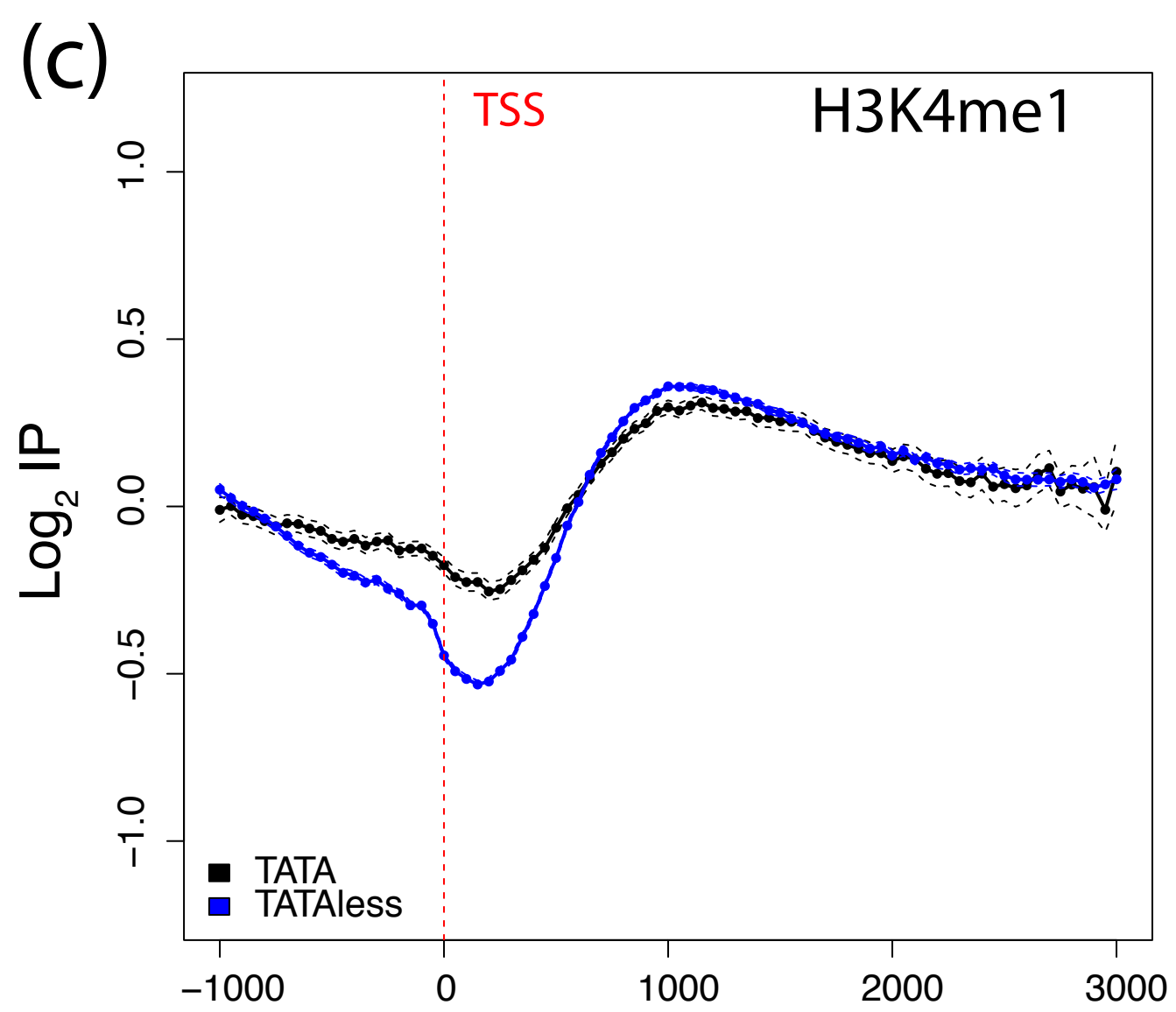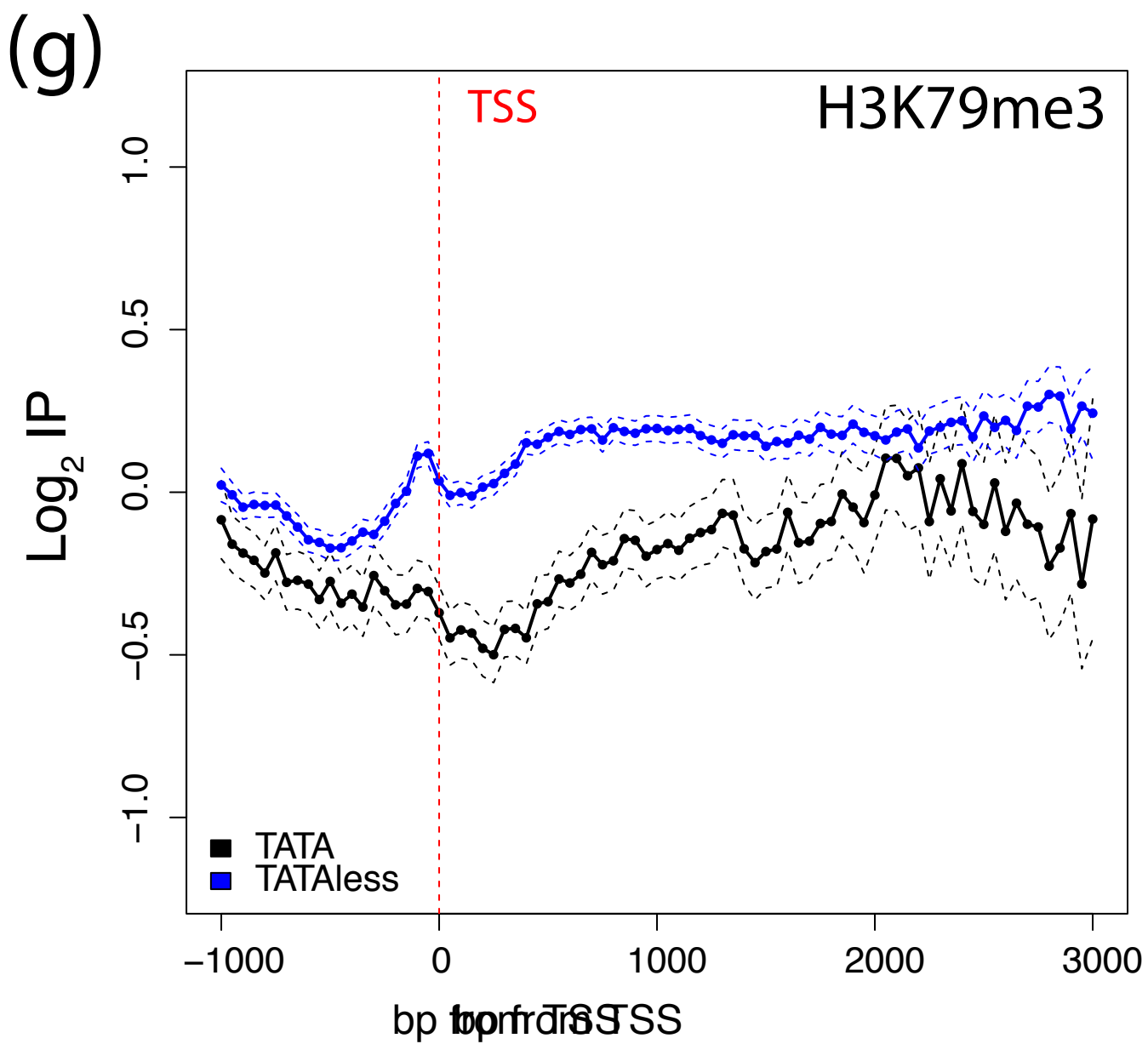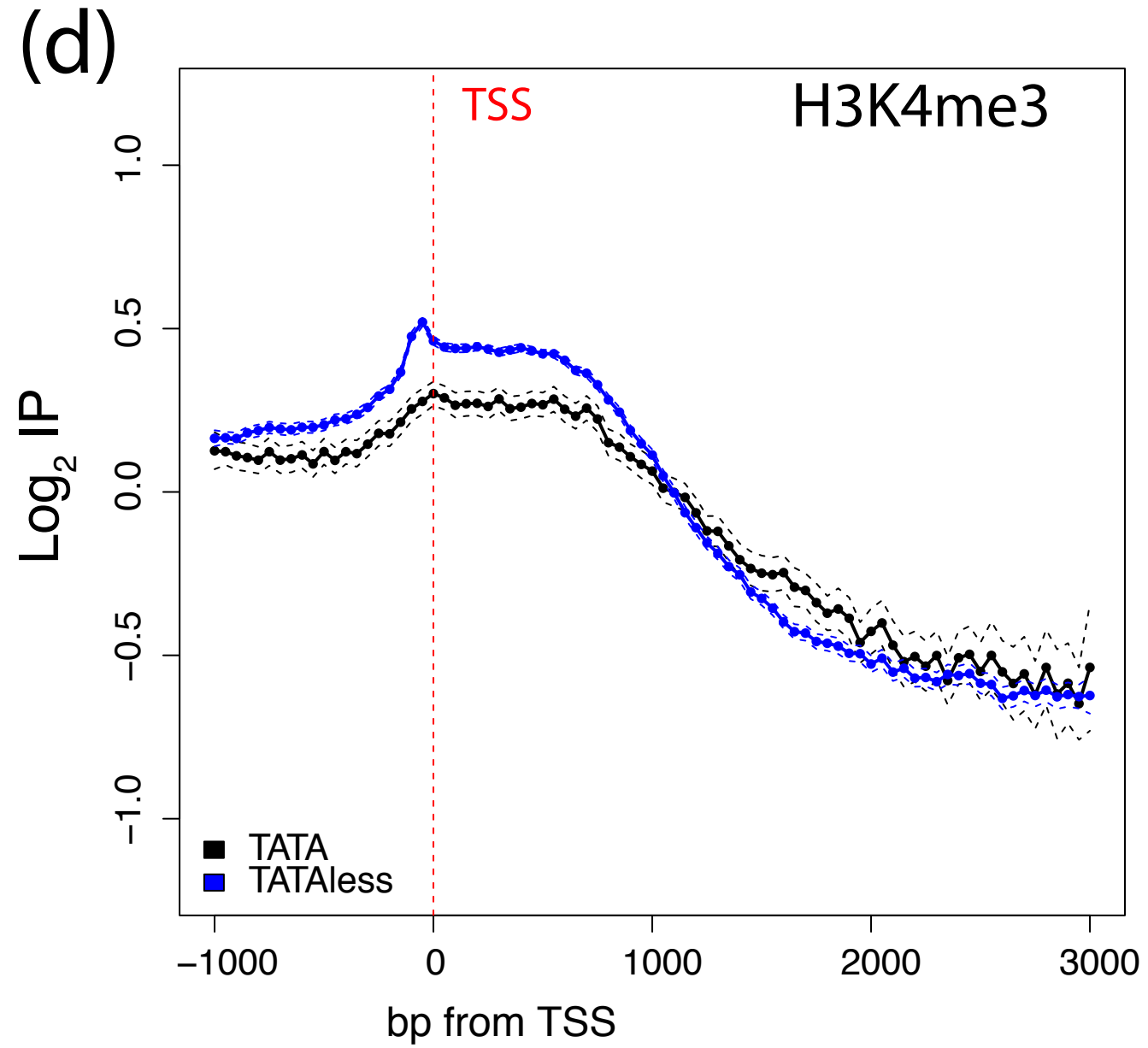

Supplement: Supplementary file 3 — Additional file 3: Figure S3: The profiles of H3K14ac (a), H4K8ac (b), H3K4me1 (c), H3K4me3 (d), H3K36me3 (e), H3K79me2 (f) and H3K79me3 (g) in relation to the levels of core histone H3 (a, c, d, e, f, g) or H4 (b) of the genes grouped according to presence or absence of a TATA box on the gene. (PDF 1 MB) [file 12864_2013_5905_MOESM3_ESM.pdf]

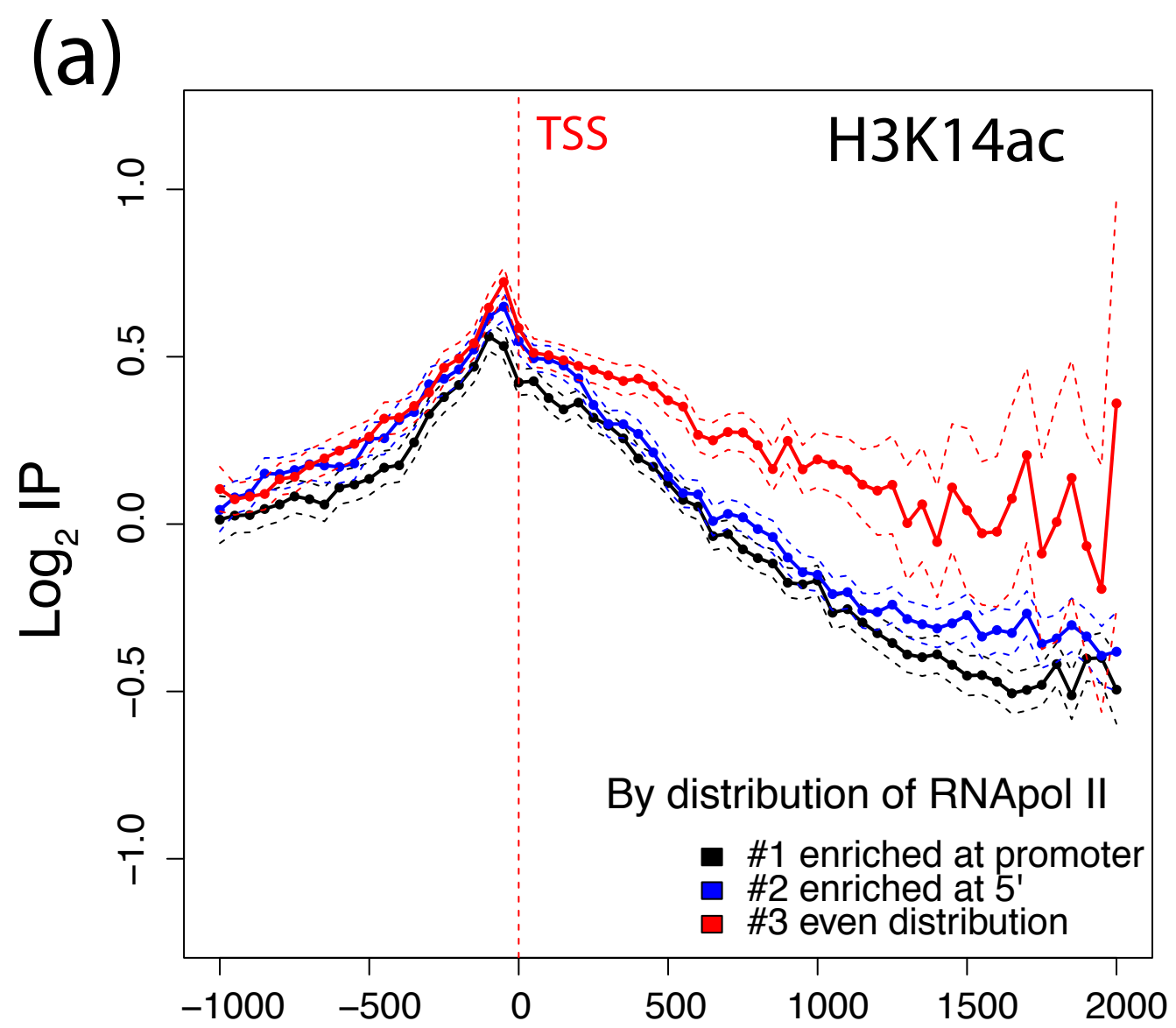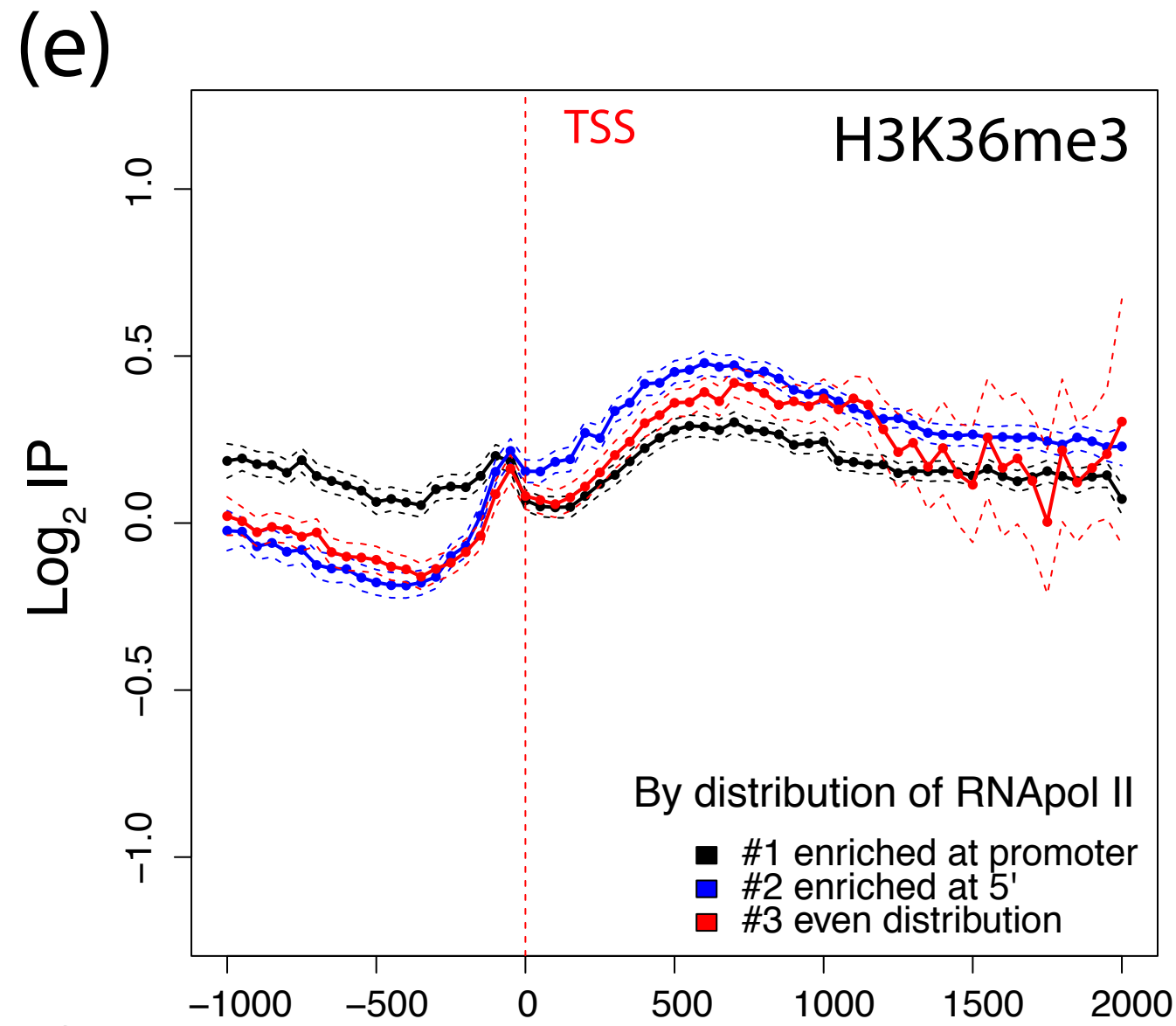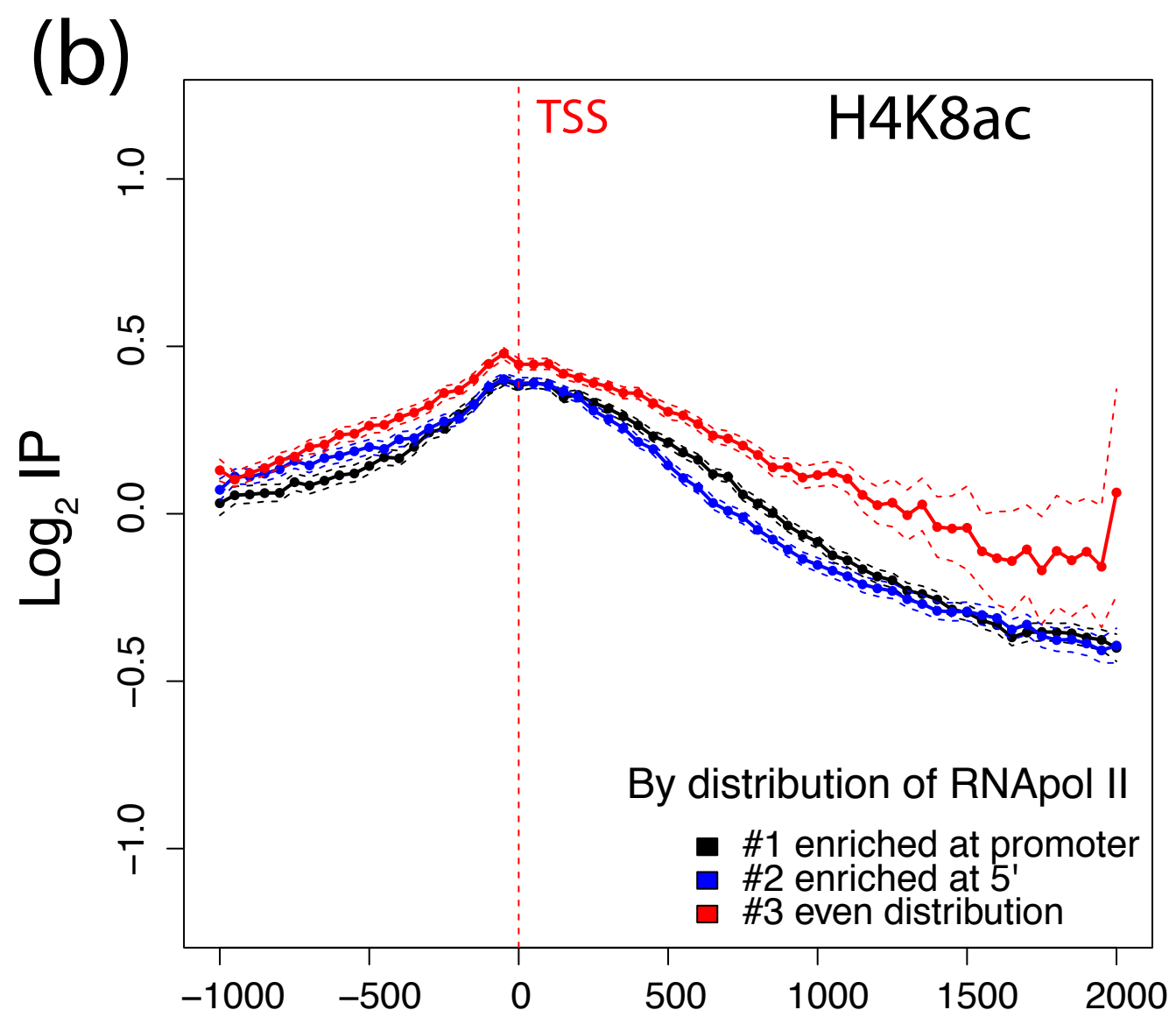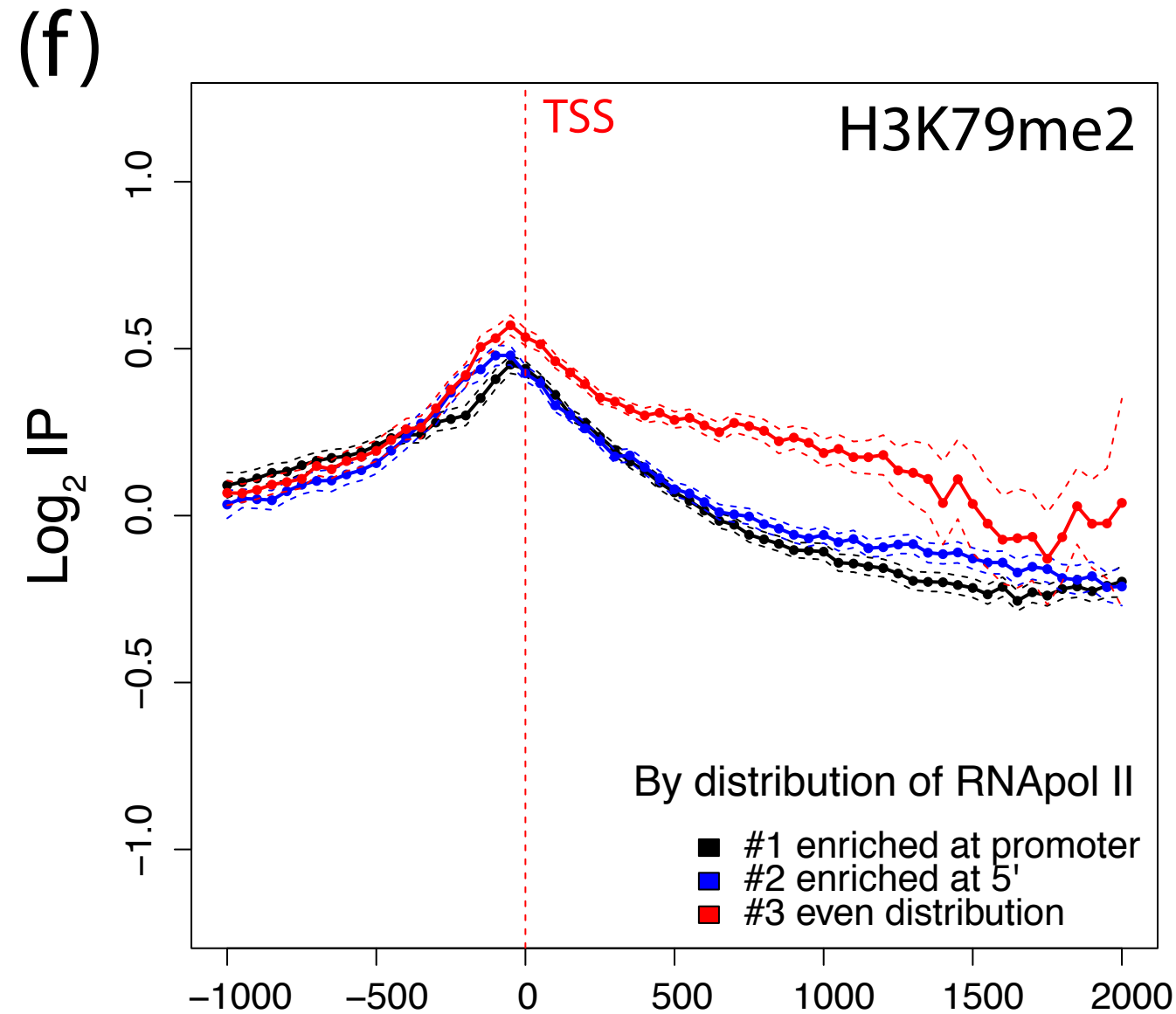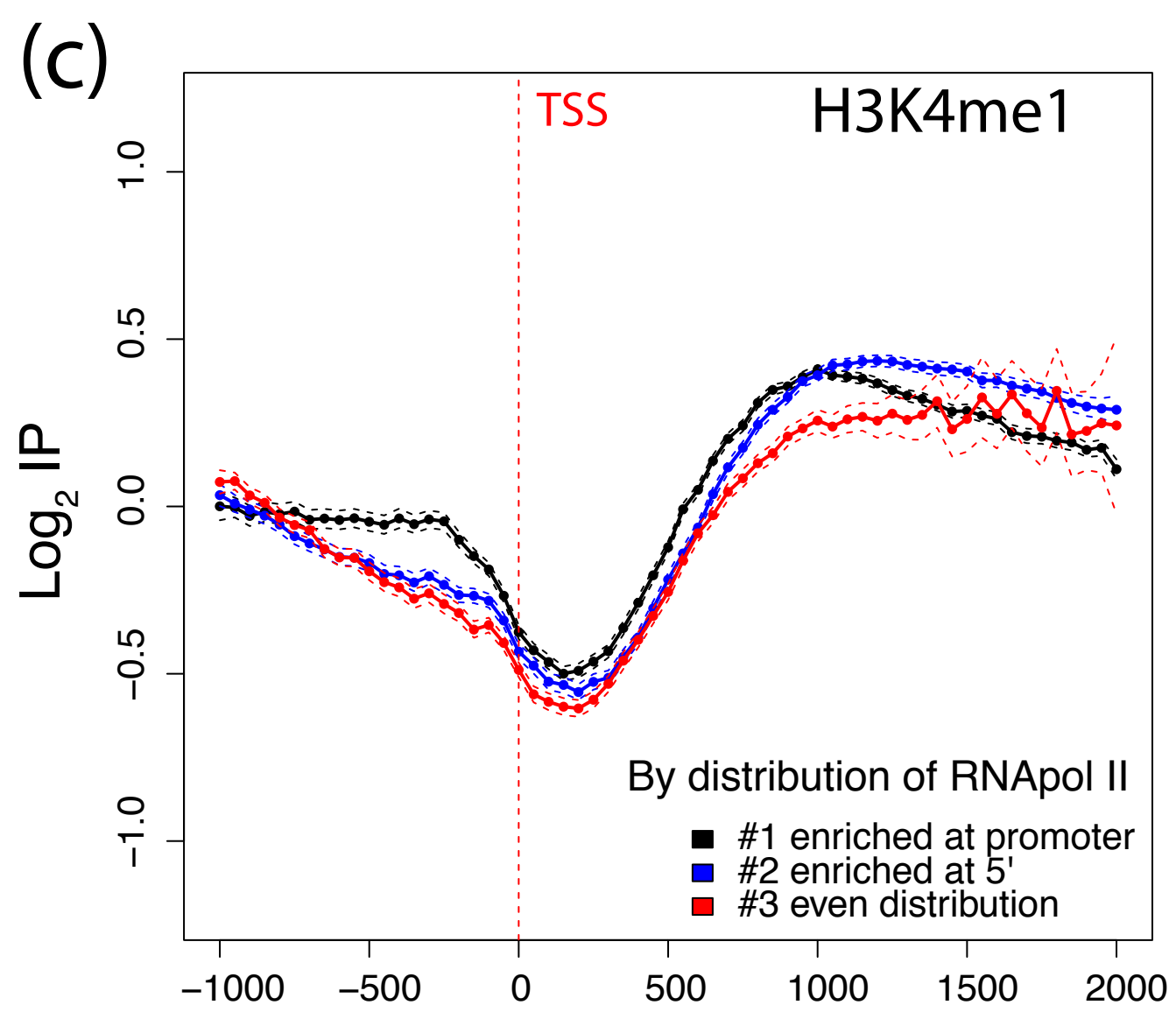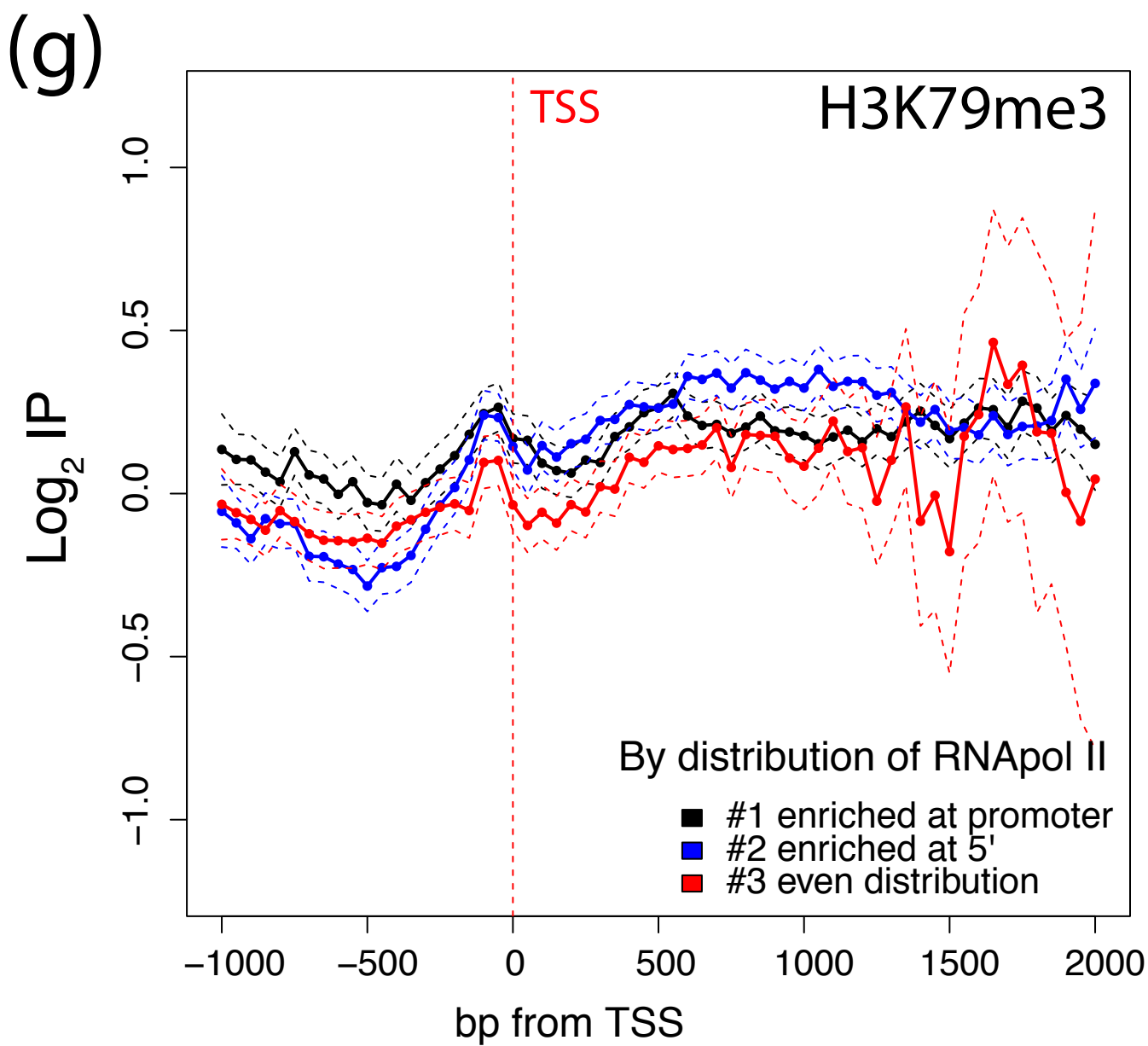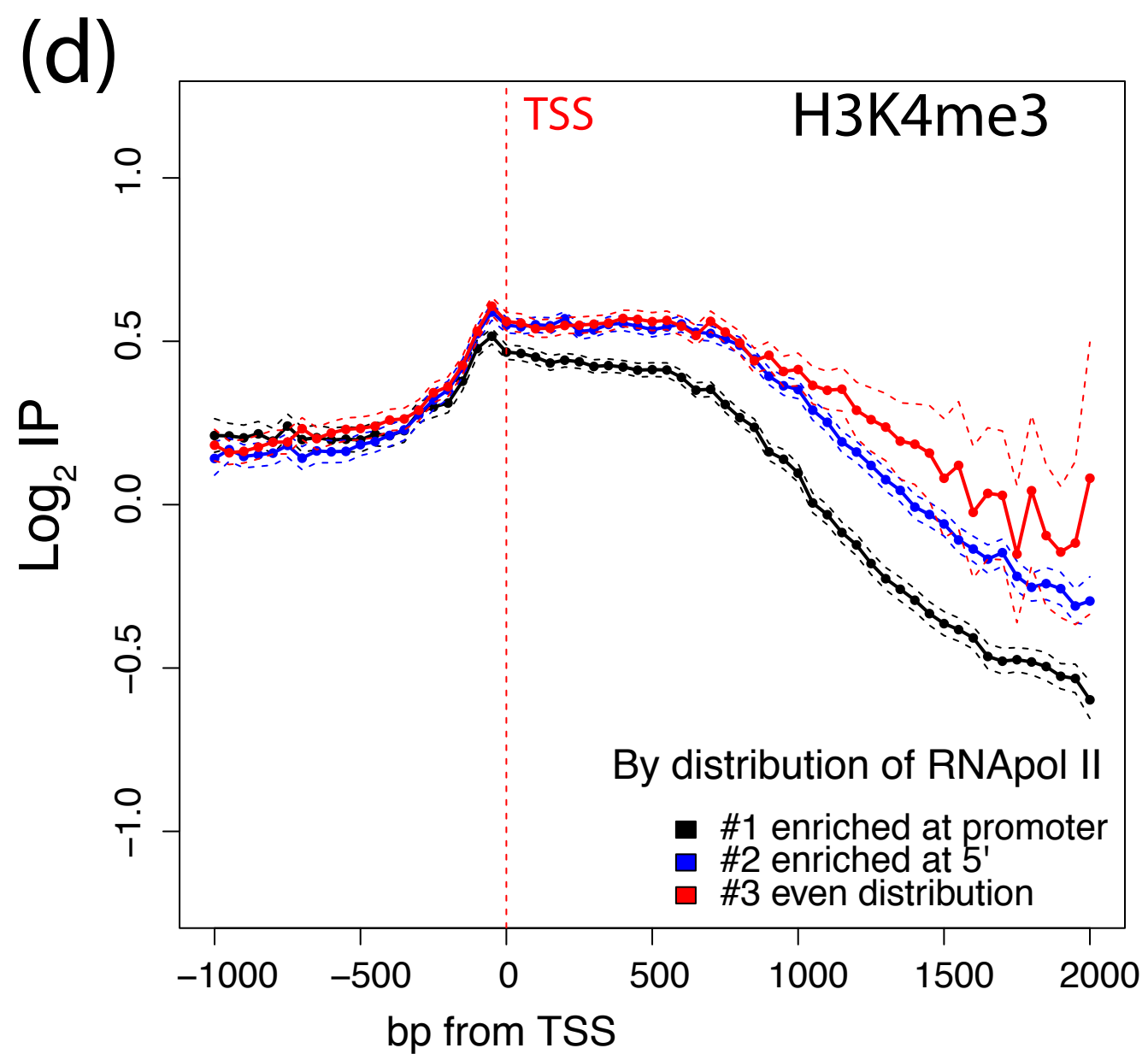

Supplement: Supplementary file 4 — Additional file 4: Figure S4: The profiles of H3K14ac (a), H4K8ac (b), H3K4me1 (c), H3K4me3 (d), H3K36me3 (e), H3K79me2 (f) and H3K79me3 (g) in relation to the levels of core histone H3 (a, c, d, e, f, g) or H4 (b) of the genes grouped according to the distribution of RNA pol II [46] on the gene. (PDF 1 MB) [file 12864_2013_5905_MOESM4_ESM.pdf]
